# Testing hypotheses of a coevolutionary key innovation reveals a complex suite of traits involved in defusing the mustard oil bomb

**DOI:** 10.1073/pnas.2208447119

**Published:** 2022-12-12

**Authors:** Yu Okamura, Hanna Dort, Michael Reichelt, Kalle Tunström, Christopher W. Wheat, Heiko Vogel

**Affiliations:** ^a^Department of Entomology, Max Planck Institute for Chemical Ecology, Jena 07745, Germany; ^b^Department of Insect Symbiosis, Max Planck Institute for Chemical Ecology, Jena 07745, Germany; ^c^Department of Biological Sciences, Graduate School of Science, University of Tokyo, Tokyo 113-0033, Japan; ^d^Department of Zoology, Stockholm University, Stockholm SE-10691, Sweden; ^e^Department of Biochemistry, Max Planck Institute for Chemical Ecology, Jena 07745, Germany

**Keywords:** plant–insect interactions, coevolution, glucosinolate–myrosinase complex, insect counteradaptation, CRISPR-Cas9 gene editing

## Abstract

Coevolutionary interactions are responsible for much of the Earth’s biodiversity, with key innovations driving speciation bursts on both sides of the interaction. One persistent question is whether macroevolutionary traits identified as key innovations accurately predict functional performance and selection dynamics within species, as this necessitates characterizing their function, investigating their fitness consequences, and exploring the selection dynamics acting upon them. Here, we used CRISPR-Cas9 mediating nonhomologous end joining (NHEJ) in the butterfly species *Pieris brassicae* to knock out and directly assess the function and fitness impacts of nitrile specifier protein (*NSP*) and major allergen (*MA*). These are two closely related genes that facilitate glucosinolate (GSL) detoxification capacity, which is a key innovation in mustard feeding Pierinae butterflies. We find *NSP* and *MA* are both required for survival on plants containing GSLs, with expression differences arising in response to variable GSL profiles, concordant with detoxification performance. Importantly, this concordance was only observed when using natural host plants, likely reflecting the complexity of how these enzymes interact with natural plant variation in GSLs and myrosinases. Finally, signatures of positive selection for *NSP* and *MA* were detected across *Pieris* species, consistent with these genes’ importance in recent coevolutionary interactions. Thus, the war between these butterflies and their host plants involves more than the mere presence of chemical defenses and detoxification mechanisms, as their regulation and activation represent key components of complex interactions. We find that inclusion of these dynamics, in ecologically relevant assays, is necessary for coevolutionary insights in this system and likely others.

## Significance

Plants and insects are locked in a coevolutionary battle, where plants develop novel chemical defenses and insects adapt to overcome them. After mustards evolved defensive compounds, butterflies evolved the ability to detoxify said compounds and then rapidly diversified. Here, we use CRISPR-Cas9 gene knockouts to remove a Pierine butterfly’s ability to detoxify mustard defensive chemistry. By assessing these gene knockouts in caterpillars on relevant plants, we find that detoxification gene expression and performance are tailored to specific plant chemical defenses. Additionally, we find evidence that natural selection acted upon these detoxification genes in several butterfly species. Our results highlight the complexity of coevolutionary interactions and reveal the key roles that detection, activation, and regulatory mechanisms play in generating specific responses.

From beetles to butterflies, plant-feeding insects account for around a quarter of all known macroscopic animals (1, 2). This vast diversity is thought to have been fueled by diffuse coevolutionary interactions with host plants, with key innovations driving speciation bursts and trait escalation on both sides of the plant–insect interaction (3, 4). However, our understanding of plant–insect systems still lacks strong mechanistic connections between gene regulation and function, microevolutionary processes, and macroevolutionary patterns, as these schools of research are rarely integrated in the testing of reciprocal hypotheses (5). Such integration is important for understanding the origins and evolutionary dynamics of coevolutionary key innovations, as such innovations are often complex phenotypes that arise from many interacting genes and their fine-tuned regulation. To fill this gap and advance the study of coevolutionary key innovations, here we use an ecological and evolutionary functional genomics approach (6) to test diverse predictions that emerge from studies of the coevolutionary interactions between Brassicales plants and their Pierinae butterfly parasites.

Pierinae butterflies (Lepidoptera: Pieridae) have been studied for over a century (7) due to their ability to overcome the glucosinolate (GSL) defenses of their host plants in the order Brassicales. A defining trait of these plants is their two-component, activated chemical defense system, known as the mustard-oil bomb (8). The inactive bomb is comprised of the enzyme myrosinase and GSL compounds, which are stored separately from each other until activation (9). Upon tissue damage (e.g., by larval feeding), myrosinase hydrolyzes GSLs, producing diverse breakdown products (10, 11). For most insects, the breakdown products of the GSLs found in Brassicales act as strong feeding deterrents, greatly reducing fitness and survival (12, 13). In contrast, Pierinae larvae are largely unharmed by GSL defenses [(13, 14), but see ref. 15] and in fact, GSL compounds have generally been coopted as stimulants for larval feeding (7, 16–20) and female oviposition (20–25)

An important component of Pierinae’s resistance to GSL defenses is conferred by larval gut-expressed proteins known as nitrile-specifier proteins (NSPs), which interact with myrosinases to divert the breakdown of GSL defenses away from the formation of highly toxic isothiocyanates (ITCs) to less-toxic nitriles instead (10, 14). Nearly 50 y ago, Ehrlich and Raven hypothesized that this ability of Pierinae butterflies to detoxify GSLs was a coevolutionary key innovation (3). The identification of NSPs as GSL-detoxification mechanisms was a major step forward in testing this hypothesis, providing a phenotype and gene for analyses (14). The birth of *NSP* and its enzymatic activity appears to have evolved shortly after basal Pierinae butterflies colonized Brassicales plants, with this colonization associated with an increased speciation rate in Pierinae (26). Subsequent study focused upon escalations in both the GSL compounds and the detoxification potential of *NSP* genes (27). In Brassicales, two major bouts of escalation in GSL complexity were associated with increased diversification rates, with the final bout giving rise to the Brassicaceae, the most speciose and GSL-diverse family of Brassicales. Analysis of detoxification evolution in Brassicales-feeding Pierinae revealed *NSP* to be part of a small gene family undergoing extensive gene birth–death dynamics. Importantly, the two lineages that independently colonized Brassicaceae used different *NSP* loci for detoxification and both lineages had increased species diversification rates compared with other lineages (27). Thus, the study of NSP gene function provides insights into the evolution of a key innovation.

The *NSP*-like gene family consists of *NSPs*, major-allergen (*MA*) proteins, and the single-domain major-allergen (*SDMA*) proteins from which the two previous genes evolved via exon duplications at the base of the Pierinae (27, 28). Several lines of evidence suggest that both *NSP* and *MA,* but not the *SDMA* genes, have played a key role in Pierinae butterfly adaptation to host plant GSL defense, leading to the following four predictions.

## *NSP* and *MA* Are Necessary for GSL Detoxification, but *SDMA* Is Not.

Whereas the GSL detoxifying capacity of NSP has been clearly demonstrated in *Pieris* species (14), the same cannot be said for MA. Previous work in *Pieris* species using heterologously expressed proteins found nitrile-forming activity only for NSP but not for MA, leading to the conclusion that MA was inactive against the tested GSLs (27). However, subsequent attempts to express functional NSP and MA proteins have been extremely challenging, to the point that we now conclude that absence of nitrile formation by heterologously expressed proteins should not be used to infer the absence of this enzymatic capacity (for more discussion refer to *SI Appendix*, Text 16). MA is a known GSL detoxifying protein in other Pieridae (27), and experiments using *Pieris melete* have shown that *NSP* and *MA* expression is only activated by the presence of GSLs (29). This suggests that both proteins may have detoxification function within *Pieris*. Furthermore, Pierinae lineages that have stopped feeding upon plants with GSLs have lost both *NSP* and *MA*, but not *SDMA,* suggesting that the former two genes are costly to maintain in the absence of GSL (26, 27).

## *NSP* and *MA* Differ in Function in Relation to GSL Variation among Plants.

While some functional differences have been identified between *MA* and *NSP*, studies have mostly been limited to in vitro assays of gut extracts and have lacked a clear ecological context. However, *NSP* and *MA* have been found to be differentially expressed when *P. melete* larvae are reared on different natural host plants, suggesting that the specificity of *NSP* and *MA*’s expression may be in response to different GSL classes (29). No such expression changes have been found in *SDMA* (29).

## MA Detoxifies a Broader Range of GSLs Compared with NSP.

This prediction is supported by the loss of *NSP*, but continued presence of *MA* in the Brassicaceae-feeding *Anthocharis* genus (27). Since *Anthocharis* feed upon host plants with diverse GSLs (30), MA likely has a broader detoxification function against GSLs compared with NSP. It has also been observed that within a butterfly species, the expression of *MA* appears to be induced by a broader range of GSLs than *NSP* (29).

## *NSP* and Potentially *MA* Commonly Experience Positive Selection.

The aforementioned studies suggest that these genes play an important and ongoing role in mediating plant–insect interactions over evolutionary time, leading diverse studies to use molecular tests of selection to try detecting evidence of these dynamics. Using consensus sequences of species in codon-based tests of selection, signatures of positive selection in *NSP* and *MA*, but not *SDMA* genes, have been identified among divergent taxa with dN/dS > 1 (27, 31), while other analyses find only an increased dN/dS rates at *NSP* among *Pieris* species though the rate is still < 1, consistent with either relaxed constraint or bouts of positive selection (27, 28, 31). Using samples of many individuals within species, two microevolutionary studies of *NSP-*family genes have been conducted to date. In the first of these candidate gene studies (32), no evidence of positive selection on *NSP* or *MA* was found in populations of *P*ieris* rapae*. However, *NSP* had an exceptionally high number of nonsynonymous fixations compared with other genes, suggestive of positive selection (32). The second study (33) found evidence of positive selection in the *NSP* genes of Japanese *Pieris napi* and suggestions of balancing selection at *NSP* in *P. melete*, with no clear evolutionary trends detected at *MA*.

In sum, while there are suggestions of positive selection at *NSP* and occasionally at *MA* among *Pieris* taxa, these variable results among combinations of taxa and methods complicate drawing conclusions from these findings. Additionally, while much of this variation is expected, as such tests detect departures from neutrality in different ways and over different time scales, their implementation has also varied in ways known to degrade estimates of positive selection (*SI Appendix*, Text 13). The codon-based tests used few taxa and were thus very underpowered (27, 28, 31), the population analyses used incomplete gene sequences for population analyses of *NSP* and *MA* (32, 33), and all these analyses failed to account for underlying population dynamics. We predict that using advances in molecular tests of selection that leverage the power of genome scale data, while accounting for demographic history, will help resolve these mixed findings.

**Fig. 1.**
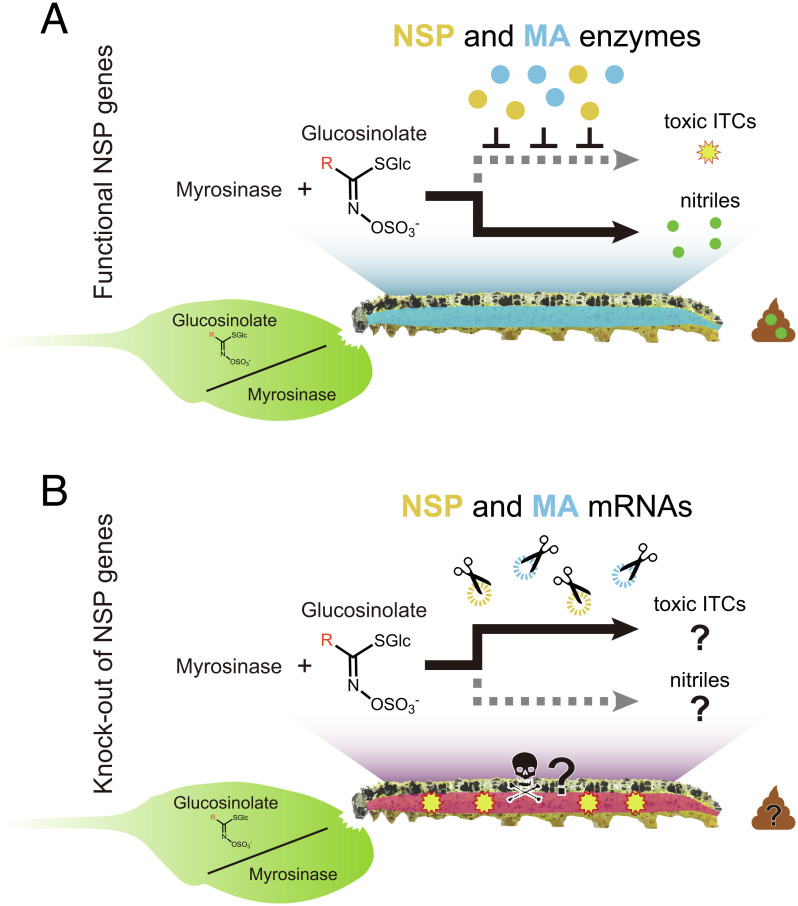
(*A*) In wild *P. brassicae* caterpillars, feeding on a GSL-myrosinase-defended plant causes no damage to the larva, as specific enzymes in the larval gut (NSP and MA) divert the breakdown of GSL compounds so that nitriles are formed instead of toxic ITCs. These nitriles are easily detectable in larval frass. (*B*) In this study, we stopped *P. brassicae*’s adaptive diversion of the GSL breakdown process by knocking out both NSP and MA genes with the CRISPR-Cas9 system. As a result of these KOs, we predicted toxic ITCs would form instead of nitriles, and KO larvae feeding on GSL-defended plants would be unable to survive.

Here we tested the above predictions to better understand how key innovations work and evolve. We used CRISPR-Cas9 mediating nonhomologous end joining (NHEJ) to knock out the function of both *NSP* and *MA*. This enabled us to characterize detoxification performance, assess functional redundancy, and understand the fitness consequences of each protein in vivo (Fig. 1). We ultimately found that NSP and MA both play important, but different roles in diverting the breakdown of GSLs and that there is a striking concordance between their induction by, and ability to detoxify, different GSLs. Additionally, we found evidence of positive selection acting on both *NSP* and *MA* genes within species lineages, albeit at different strengths, consistent with the ongoing importance of these genes to their lineages’ evolutionary success. Our work represents a major step forward both for the study of the Pierinae–Brassicales system and for the field of coevolutionary biology; we demonstrate that the genes involved in a key innovation phenotype experience positive selection at a microevolutionary scale, with their regulation fine-tuned to produce plastic responses for host plant-specific detoxification.

## Results and Discussion

### Building a Model System for Testing GSL Detoxification.

To test the in vivo functional role of *NSP*-like gene family members, we chose to develop *P*ieris* brassicae* as a lab model, as it lays eggs in large clutches and is easily reared in continuous generations. We sequenced and assembled the genome of a wild-type (WT) male *P. brassicae* individual (n = 87 contigs, N50 = 18.5 Mbp, totaling 265 Mbp in size) and generated a genome annotation (n = 16,334 genes; *SI Appendix,* Table S2), which revealed two recently duplicated copies of the *NSP* genes on the same contig (*SI Appendix,* Fig. S1*B*), separated by a 4.8-kb spacer region and differing in only six nucleotides along their 1,896-bp coding region (*SI Appendix,* Fig. S1*B*). Only one copy of *MA* was found, which was located on a different chromosome from *NSP*, as in the closely related species, *P. napi* (34) (*SI Appendix,* Fig. S1*A*). Genome sequencing and annotation methods are detailed in *SI Appendix*, Texts 3 and 4.

### *NSP* and *MA* Expression, but Not *SDMA* Expression, Is Affected by Host Plant Chemistry.

**Fig. 2.**
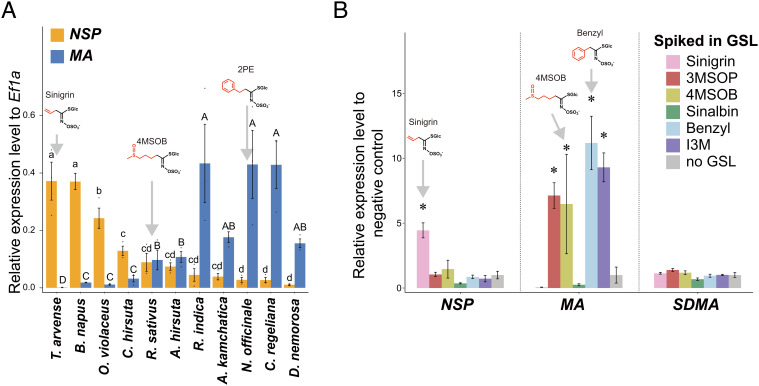
(*A*) Relative expression levels of *NSP* and *MA* compared with a control gene (*Ef1a*) in WT *P. brassicae* larvae reared on different host plants. Here, larvae feeding on *T. arvense* (a species with high sinigrin concentration) saw the greatest upregulation of *NSP*, whereas larvae feeding on *N. officinale* [a species with high benzylic GSL concentration (2PE: 2-phenylethyl GSL; gluconasturtiin)] experienced the greatest upregulation of *MA*. Different letters on each box show significance (pairwise *t* test on log-transformed expression values, false discovery rate (FDR) corrected *P* ≤ 0.05). (*B*) Relative expression levels of *NSP*, *MA*, and *SDMA* in WT *P. brassicae* larvae following feeding on mutant *A. thaliana* lines supplemented with various GSL compounds. Here, *NSP* was significantly up-regulated in response to sinigrin, and *MA* was significantly up-regulated in response to benzyl-GSL, I3M-GSL, 3MSOP-GSLs, and 4MSOB-GSLs. [ANOVA to the negative control (no GSL) on log-transformed expression values, "+": FDR corrected *P* ≤ 0.05.]

Since previous work found different expression of *NSP* and *MA*, but not *SDMA*, in the guts of *P. melete* larvae that fed on host plants differing in GSL profiles (29), we sought to both clarify this dynamic and verify this response in *P. brassicae* prior to gene editing. When we fed *P. brassicae* larvae on 11 different, ecologically relevant Brassicaceae host plant species, *NSP* and *MA* showed different expression patterns, which were commonly complementary (Fig. 2*A*). *NSP* was primarily up-regulated in response to plants with high concentrations of aliphatic GSL, such as sinigrin-rich *Thlaspi arvense* (35, 36). *MA* was up-regulated in response to plants with high benzylic GSL concentrations, such as *N*asturtium* officinale*, which is rich in gluconasturtiin (36, 37). Our observations in *P. brassicae* are consistent with those for *P. melete* in finding statistical support for the differential regulation of *NSP* and *MA* in response to diverse host plants, lending further support to other preliminary observations of differential *NSP* and *MA* regulation in response to hostplant differences in four species of *Pieris* butterflies (29). Together these studies strongly suggest that, in general, these two genes are regulated differently in response to variable GSL profiles that larvae encounter in their host plants. However, assessing whether GSL profiles alone induce differential expression of *NSP* and *MA* is challenging, as plant species differ in myriad ways.

Next, we directly tested whether the expression of *NSP* and *MA* differs as a response to specific GSL compounds when feeding on a standardized host plant: an *Arabidopsis thaliana* quad-mutant line that contained no GSLs, hereafter referred to as GSL-null *A. thaliana*. Mutations in this GSL-null line reduced indole GSL and camalexin content (*cyp79B2* and *cyp79B3*), as well as aliphatic GSL content (*myb28* and *myb29*). Following the methods of Schramm et al*.* (38), we infiltrated detached GSL-null rosette leaves with aqueous solutions of one of six individual GSLs, resulting in leaves with ecologically relevant concentrations of a given GSL (*SI Appendix*, Fig. S4). GSLs used for treatments were chosen due to their presence in host plants commonly used by *Pieris*. Neonate larvae were fed on treated leaves, or on untreated, control leaves for 5 d, at which time, qPCR was used to measure expression of our target genes. Using this setup, we were able to directly examine the effects of specific GSL profiles on larvae while avoiding correlated changes in plant phenotypes.

The *NSP* gene was uniquely up-regulated only in larvae fed with sinigrin, while *MA* was highly expressed only when the larvae were fed with 3-methylsulfinylpropyl (3MSOP), 4-methylsulfinylbutyl (4MSOB), benzyl- or indol-3-yl-methyl (I3M) GSLs (Fig. 2*B*), indicating the latter’s broader induction response. Both of these results were consistent with previous findings on natural plants (Fig. 2*A*). In contrast, *SDMA* was not significantly induced by any GSLs (Fig. 2*B*). In sum, in a constant plant background, larvae can detect and discern among GSLs. Moreover, GSL variation alone is sufficient to induce a fine-tuned expression response of *NSP* and *MA* in *P. brassicae* larvae, suggesting that both are important in different ways for GSL detoxification. Two nonmutually exclusive hypotheses for alternative expression of *NSP* and *MA* are that they a) differ in their detoxification performance against GSL compounds, consistent with their expression patterns, and b) interfere with each other’s performance. Both hypotheses predict concordance between GSL-specific induction and detoxification capacity, with genes being induced when they are needed.

### Proxy Assays Using CRISPR-Cas9 KOs Reveal NSP and MA Are Necessary for Detoxification of Aliphatic and Benzylic GSLs.

To test for differences in *NSP* and *MA* detoxification performance, we used CRISPR-Cas9-mediating NHEJ to create three germline, knockout (KO) lines of *P. brassicae*: a *NSP*-KO line (Δ*NSP*; knocked out both of two *NSP* copies), a *MA-*KO line (Δ*MA*), and a double-KO line (Δ*NSP*Δ*MA*; knocked out both of two *NSP* copies) lacking both *NSP* and *MA* gene function (*SI Appendix,* Figs. S2 and S3). Loss of *NSP* and *MA* expression in these KO lines was confirmed through qPCR (*SI Appendix,* Fig. S2) and fecal content assays that quantified GSL detoxification function (*SI Appendix,* Fig. S5), with the absence of off-target mutations confirmed via whole-genome sequencing of all three KO lines.

**Fig. 3.**
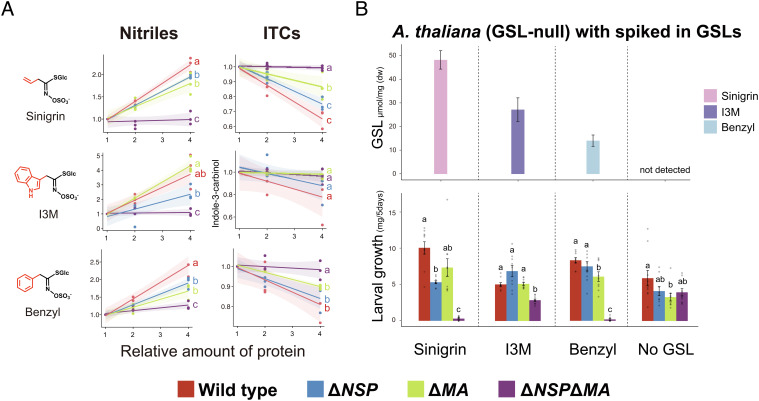
(*A*) Results from the NSP/MA activity assay on different concentrations of the larval gut extract. Here, the values on the X-axis represent the concentration of *P. brassicae* gut protein used (1x, 2x, or 4x), with 1x being 0.5 µg/µl gut protein in 50-µl 2-(n-morpholino)ethanesulfonic acid (MES) buffer. Three replicates per line of larvae were prepared for each concentration. On the Y-axis, for each of three GSL compounds, we report the amounts of nitriles and ITCs detected postassay, relative to the amounts found when using 1x concentrations of the larval gut from the same line. These results demonstrate that Δ*NSP*Δ*MA* gut extracts are much less efficient at blocking ITC formation from GSLs than both WT and single-KO gut extracts. Furthermore, slight activity differences exist between Δ*NSP* and Δ*MA* in nitrile formation of I3M-GSL, and in ITC formation of sinigrin. Statistically significant differences (pairwise *t* test: FDR corrected *P* ≤ 0.05) are represented by different letters. (*B*) Results from the GSL spike-in feeding assay. The GSL profiles of quad-GSL *A. thaliana* with GSL spike-ins are reported in the *Top* panels, and the mean larval growth rates of the four *P. brassicae* lines fed on each spike-in plant are reported in the *Bottom* panels. Ten larvae per lineage were used for each treatment group.

To assess the direct impacts of specific classes of GSLs on the *P. brassicae* KO lines, we used two functional assays that are proxies of natural plant–insect interactions. First, we assessed the ability of larval gut extracts to convert different GSL compounds to inert (nitriles) vs. toxic (ITC) products in a series of in vitro enzyme activity assays (described in *SI Appendix*, Text 10). These were followed by larval feeding assays, where WT, Δ*NSP*, Δ*MA*, and Δ*NSP*Δ*MA P. brassicae* were fed on leaves of GSL-null *A. thaliana* mutants supplemented with different GSLs (Fig. 3). Compared with all other lines, Δ*NSP*Δ*MA* lines experienced a near complete loss of nitrile-forming activity and a high level of ITC production (Fig. 3*A* and *SI Appendix,* Fig. S6), as well as a significantly lower larval growth and survival on all GSL-treated plants (Fig. 3*B*). Having either NSP or MA significantly increased nitrile concentration and larval growth compared with Δ*NSP*Δ*MA* lines, consistent with the nitrile-specifying activity expected of NSP, and now documented here for *Pieris* MA as well.

We next focused on detecting functional differences between these two detoxification enzymes. First, in vitro activity assays detected no clear difference between Δ*NSP* and Δ*MA* lines in response to either sinigrin or benzyl-GSL exposure. Although there were significant differences in ITC production between lines fed on sinigrin, these results were opposite to our expectations based on the expression patterns of *NSP* and *MA*, and feeding assay with sinigrin supplied plants (Fig. 3 *A* and *B*). There was little difference in the growth rate of these two single-KO lines, as both experienced significantly lower growth than WT larvae in response to sinigrin, with benzyl-GSL results less clear though in the same direction (Fig. 3*B*). Further complicating the interpretation of the single-KO results was their varied response to I3M-GSL treatments. Gut tissue from Δ*MA* lines produced significantly more nitriles than tissue from Δ*NSP* lines (Fig. 3*A*), which might indicate that NSP is a more effective detoxifier of indole GSLs than MA. However, Δ*NSP* lines experienced even higher rates of larval growth in the feeding assays on GSL-supplemented plants, indicating the exact opposite (Fig. 3*B*). Although we performed this experiment cohort based due to technical limitations and the result should be interpreted in the light of this experimental design, one potential explanation for this discrepancy might be that neither protein is necessary for indole GSL feeding. This hypothesis is supported by the larval growth rates in Δ*NSP*Δ*MA* lines, where larvae lacking both *NSP* and *MA* could not survive on *A. thaliana* supplemented with sinigrin or benzyl-GSL but could survive on I3M-GSL-treated plants (Fig. 3*B*). These results suggest that while MA and NSP can convert I3M-GSL to nitriles, a general stress response mechanism is sufficiently effective to support larval growth in the absence of MA and NSP. We find this result very exciting, as indolic GSLs likely were one of the ancestral GSLs that pierid butterflies first encountered when they first shifted their feeding on to Brassicales plants (27). However, phenylalanine-derived benzyl-GSLs were likely also present in these ancient Brassicales (27), and benzyl-GSLs are lethal in the absence of NSP and MA (Fig. 3*B*), suggesting there remains much to be learned about the initial colonization of Brassicales by pierid butterflies. While colonization of novel plant lineages is expected to be aided by general detoxification mechanisms (39), and our findings of a general detoxification mechanism only apply for a subset of a larger suite of GSLs likely involved, these findings will help focus future studies of Brassicales colonization dynamics.

In sum, results from our two proxy assays allow us to infer that NSP and MA proteins are necessary for *Pieris* survival on plants containing aliphatic or benzylic GSLs, but not indolic GSLs (I3M-GSL). However, we could not detect robust differences between the enzymatic function of NSP and MA in the in vitro assay with the gut extract. One reason for this might be that in both of the proxy assays used, the combination of myrosinase and GSLs did not reflect their evolutionary history of interactions within plants. Myrosinase enzymes form a small gene family, wherein plant species have multiple gene copies and copy number varies from between 3 and 10 copies per species (40, 41). Additionally, orthologous and paralogous versions of myrosinase can differ in myriad ways, including their substrate specificity, amount, and the rate at which they convert GSLs into toxic ITCs depending on the plant GSL profile (42, 43). There are also a number of additional specifier proteins that can affect the fate of GSL breakdown, and those can potentially interact with NSP and MA and modify their activity (10, 44). Hence, the proxy assays we used, which are common in this type of work, not only simplified a complex dynamic, but brought together combinations of myrosinases and GSLs that may not be reflective of natural systems in important ways. Thus, while such assays are suitable for coarse-grained activity detection, they may intrinsically limit the detection of fine scale, ecologically relevant functional differences, embodying a recent call for the need to conduct functional biology in a natural context (45). One way to test this hypothesis would be to rear our KO lines on ecologically relevant plants that differ significantly in these focal GSL compounds.

### Ecologically Relevant Assays Using CRISPR-Cas9 KOs Show Concordance between Regulation and Function of NSP Genes.

**Fig. 4.**
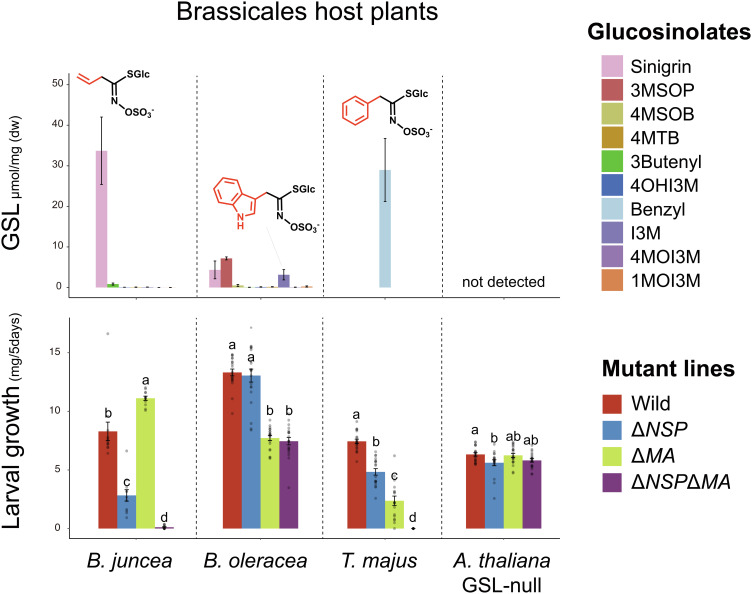
The GSL profiles of four ecologically relevant host plants used in a *P. brassicae* feeding assay (*Top*) paired with the mean larval growth rates of the four lines of *P. brassicae* larvae (WT, Δ*NSP*, Δ*MA*, and Δ*NSP*Δ*MA*) reared on each plant (*Bottom*). Significant differences in larval growth are represented by different letters (e.g., a is significantly different from b, but not ab). Twenty larvae per lineage were fed on each host plant species, for a total of 80 larvae per lineage being used in the assay. 4MTB: 4-methylthiobutyl, 4HOI3M: 4-hydroxy-indol-3-yl-methyl, 4MOI3M: 4-methoxyindol-3-yl-methyl, 1MOI3M: 1-methoxyindol-3-yl-methyl.

Given the complexity involved in creating an evolutionarily relevant assay of GSL detoxification, we conducted a second feeding assay, where KO and WT *P. brassicae* lines were fed on host plant species they commonly use in the wild (46, 47), as well as on GSL (and camalexin)-null *A. thaliana*. The chosen host plant species (*Brassica juncea*, *Brassica oleracea*, and *Tropaeolum majus*) have different GSL profiles (Fig. 4), each enriched for one of the previously assessed GSLs (Fig. 3*B*). Again, we found that the Δ*NSP*Δ*MA* line could not survive on plants enriched for sinigrin and benzyl-GSL (*B. juncea*, *T. majus*). Δ*NSP*Δ*MA* larvae did, however, survive on GSL-null plants, as well as those having I3M-GSL and generally lower levels of GSLs (*B. oleracea*) (Fig. 4).

In stark contrast to the results of the proxy assays, the single-KO lines differed significantly among these natural host plants (Fig. 4) and in a manner predicted by the gene expression results (Fig. 2). For instance, compared with WT, the Δ*NSP* line saw decreased performance on sinigrin-rich plants (*B. juncea*) (Fig. 4), consistent with *NSP* upregulation when feeding upon sinigrin-spiked GSL-null plants (Fig. 2*B*). Similarly, the Δ*MA* line performed worst on *T. majus* (Fig. 4), which is rich in benzyl-GSL, the compound that induced the highest expression of *MA* among all the test GSLs (Fig. 2*B*). Thus, the dynamic regulation of *MA* and *NSP* does accurately predict differences in their detoxification performances in response to host plant GSL content, with NSP performing better against, and induced by, aliphatic GSLs and MA similarly to benzyl-GSLs. When feeding on plants enriched in benzyl-GSL (*T. majus*), MA appears to compensate for the loss of NSP (Δ*NSP*) much better than NSP compensates for MA (Δ*MA*), with both enzymes’ function appearing to contribute additively to WT function. This suggests partial overlap in both enzymes’ function against benzyl-GSL, with MA performing much better than NSP, but without competition or interference. Finally, MA appears to have a broader detoxification capacity compared with NSP, as feeding results upon *B. oleracea* show that the Δ*MA* and Δ*NSP*Δ*MA* lines had significantly lower growth rates. Thus, conducting our functional assays in a more natural context profoundly altered what we could observe compared with proxy assays, suggesting that natural diversity in plant myrosinases and potentially other factors are an essential component of NSP-GSL coevolution.

### Signals of Positive Selection on GSL Detoxification Genes across *Pieris* Species.

The findings above provide evidence for many of our predictions, namely that NSP and MA are dynamically regulated in a way that reflects their role in GSL detoxification. Such concordance between regulation, performance, and specificity likely has arisen due to natural selection, but robust insights into the selection dynamics acting on these genes compared with the rest of the genomes are currently lacking. Our discovery of two nearly identical, tandem copies of *NSP* in *P. brassicae*, coupled with its dynamic regulation and functional performance in response to specific GSL compounds, led us to hypothesize that *NSP* in *P. brassicae* has experienced strong positive selection, both for fixation of adaptive amino acid variation, as well as subsequent tandem duplications for increased expression level. To test this hypothesis of positive selection, we estimated the strength and direction of selection on coding genes across the genome, against which we could compare the selection dynamics acting upon *NSP*, *MA,* and *SDMA*. In order to estimate selection dynamics that have occurred on the lineage leading to *P. brassicae*, and place this in the context of related species, we used the high-dimensional McDonald–Kreitman Poisson random field method (HDMKPRF) (48). HDMKPRF is a genomics-era advance upon the traditional single-gene MK test (49), as it leverages the information available from combining among gene, within-species polymorphisms and between-species divergence, from population samples of multiple species, allowing for lineage-specific estimates of N_e_, mutation rate and per-gene selection intensities (for more information on why we selected this test, refer to *SI Appendix*, Text 13). Specifically, HDMKPRF estimates a per-gene selection intensity unique to a species’ lineage since their last common ancestor in the analysis, which when significantly > or < 0 indicates positive or negative selection, allowing for the relative ranking of genes and assessment of their evolutionary history.

**Fig. 5.**
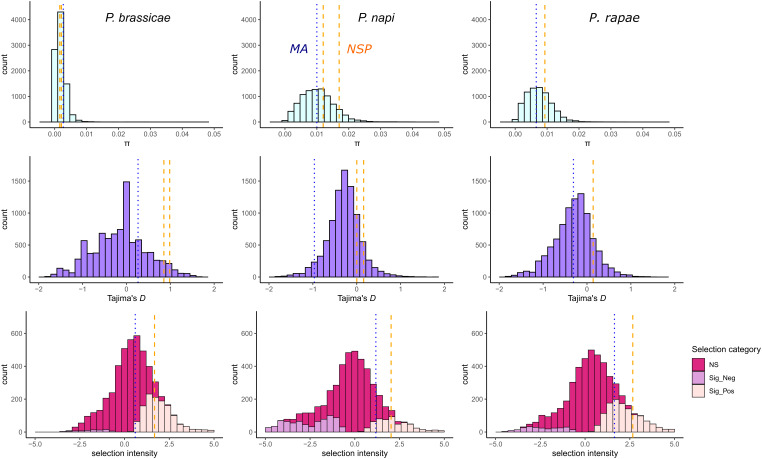
Distributions of genetic diversity and molecular tests of selection in three species of *Pieris* butterflies. Estimates of per-gene nucleotide diversity (pi, *Top*), Tajima’s *D* (*Middle*) and selection intensities (*Bottom*), are for the coding regions of single-copy orthologs (SCO) shared by three *Pieris* butterfly species (columns)**.** The locations of estimates for *NSP* and *MA* genes in each distribution are represented by orange dashed lines and blue dotted lines, respectively. Estimates were calculated for n = 4,790 SCO shared by all species. *Bottom* row depicts genes detected as undergoing selection (*P* < 0.05) in pink (negative selection) and orange (positive selection). Estimates for the selection coefficient of *NSP* and *MA* are represented in each distribution by a dashed orange and blue line, respectively.

Using a single-copy ortholog set of genes (n = 4,790) from three *Pieris* species (*P. brassicae*, *P. napi,* and *P. rapae*), as well as *NSP* sequences for each species (see *Methods*), HDMKPRF estimated the median positive selection intensity for *NSP* in *P. brassicae* of 1.675 (*P* = 0.0011), which ranked in the top 17% of selection intensities for all *P. brassicae* genes included in our analysis (Fig. 5 and *SI Appendix*, Table S7). Additionally, *NSP* was also estimated to be under positive selection in *P. napi* and *P. rapae*, with median estimated selection intensity of 2.029 (*P* = 0.0004) and 2.665 (*P* < 0.0001), respectively (Fig. 5 and *SI Appendix*, Table S7). *MA* was not found to be under positive selection in *P. brassicae*, while weak positive selection was detected in *P. napi* and *P. rapae* (*SI Appendix*, Table S7)*. SDMA* was not under directional selection in any species (*SI Appendix*, Table S7). Genome-wide estimates of selection in these *Pieris* species, with roughly 28 to 30% of genes estimated as having undergone positive selection along a given species lineage (*SI Appendix*, Table S8), are consistent with similar findings in insects with large population sizes (e.g., *Drosophila simulans, Heliconius melpomene*) (*SI Appendix*, Text 13) (50, 51). Thus, while *NSP* appears to experience stronger and more consistent positive selection than *MA*, perhaps reflecting the differences in specificity of these genes (Fig. 4), both genes are far from the most strongly selected in these species (Fig. 5).

For additional insight, we also calculated per-gene estimates of nucleotide diversity and Tajima’s *D* for this same dataset (52) (*SI Appendix*, Text 14). Neither *NSP* nor *MA* were outliers in these metrics in any species (Fig. 5). Since HDMKPFR and Tajima’s *D* detect positive selection on vastly different times scales, together they provide insights into the evolutionary dynamics along the unique lineage of each species (*SI Appendix*, Text 13). For example, in *P. brassicae, NSP* displays significant positive selection via the HDMKPRF test, but average levels of Tajimas’ *D*. This suggests that selective sweeps at *NSP* (as identified by HDMKPRF) fixed sufficiently long ago during the evolutionary history of this species that the site-frequency spectrum has returned to near equilibrium in modern populations (as identified by Tajima’s *D*). Together these results suggest the action of adaptive evolution of *NSP* and *MA* on microevolutionary time scales in extant species lineages, concordant with expectations from our previous macroevolutionary and functional insights.

## Conclusions

This study in the Pierinae–Brassicales system reveals how *NSP* and *MA* functionally differ and demonstrates the consequences for larval growth (a fitness proxy) if one or both genes are lost, in vivo. Our findings show that coevolution clearly consists of more than gene-for-gene interactions. Instead, fine-tuning of adaptive mechanisms occurs at many levels, ranging from specialization of molecular function to development of environment-dependent regulatory and activation responses. We find that these insect herbivores deploy a much more modular, context-dependent detoxification system undergoing stronger and more consistent positive selection than previously documented. Using a panoply of detection, regulatory, and detoxification mechanisms, *Pieris* butterflies accurately tailor how they defuse diverse mustard-oil bombs, displaying a sensitivity to both plant GSL profiles and their activation. Thus, this long running plant–insect war involves much more than chemical defenses and their detoxification. Our results suggest that regulation and activation represent key components of these multitrophic interactions, warranting their inclusion in future coevolutionary studies in this and other systems.

## Materials and Methods

### Expression of *NSP* and *MA* in *P. brassicae* Larvae on Different Host Plants.

We sampled *P. brassicae* egg clutches from the field in Hokkaido, Japan and reared them to adult stage on *B. oleracea*. Adults were paired by hand, and eggs were collected from fertilized females. Host plants for F1 larvae were grown from field-sampled seeds of 11 Brassicaceae species (*Arabidopsis kamchatica*, *Arabis hirsuta*, *Brassica napus*, *Cardamine hirsuta*, *Cardamine regeliana*, *Draba nemorosa*, *N. officinale*, *Orychophragmus violaceus*, *Raphanus sativus*, *Rorippa indica*, *T. arvense*) in a greenhouse at 25 °C with 60% relative humidity and L16:D8 for 7 wk. We introduced 10 F1 neonate larvae (approx. 12 h old) to each host plant species and let them feed under the same conditions. After 5 d of feeding, we measured larval weight and froze each larva with liquid nitrogen. We processed the frozen larvae with RNAlater-ICE (Invitrogen) and stored them at −20 °C until RNA extraction (*SI Appendix*, Text 5). Extracted RNA was quantified by RT-qPCR, which was performed using TB Green® Premix Ex Taq™ II (Tli RNaseH Plus) and the CFX Connect Real-Time PCR Detection System (BIO-RAD). We ran RT-qPCR with two technical replicates for each sample. Data were analyzed with ddCq methods in R (53).

### Generating NSP-KO Mutant Lines with CRISPR-Cas9.

We generated single-guide RNAs (sgRNAs) following the methods outlined in *SI Appendix*, Text 6. The Alt-R CRISPR-Cas9 System was then used with the sgRNAs to prepare Cas9:sgRNA ribonucleoprotein complexes, which were injected into fresh (<2 h old) *P. brassicae* eggs with a FemtoJet Microinjector. Injected eggs were kept in a sealed petri dish with wet tissues and incubated at 25 °C until hatching. Hatched G0 larvae were reared to adulthood on *B. oleracea* and were then genotyped and selectively hand-paired to create G1 Δ*NSP* and Δ*MA* (*SI Appendix*, Text 7 and Fig. S3). An Δ*NSP*Δ*MA* line was generated by injecting eggs of the *ΔNSP* line with Cas9:sgRNA complexes that targeted *MA* (*SI Appendix*, Text 7)*.* For all generated mutant lines, we performed RT-qPCR to measure the expression levels of the target gene, using the same methods as in our expression analyses (*SI Appendix*, Fig. S2). To minimize the effect of different inbreeding levels among the WT and KO lines, we also kept the WT line in the laboratory in parallel with the KO lines. The absence of such effects is seen in the lack of any consistent signature of inbreeding depression on larval growth rates in our KO lines under the GSL-null treatments (Figs. 4 and 5).

### Identifying Off-Target Effects of CRISPR-Cas9 Cuts.

To assess if off-target cuts were made by our CRISPR-Cas9 system, we sequenced the whole genome of one individual from each of the three KO lines with MinION and then used the Integrative Genomics Viewer (IGV) (54) to manually inspect the potential off-target gRNA-binding sites predicted by cas9off v 1.5.1 (55, 56) (*SI Appendix*, Text 7). Of the 60 off-target sites predicted for the *NSP* gRNA and the 55 sites predicted for the *MA* gRNA, we only identified one potential off-target cut in a nongenic region in the Δ*MA* line (*SI Appendix*, Fig. S8). Further inspection revealed this was an allelic variant in the founder population, as the same polymorphism existed in WT individuals (*SI Appendix*, Fig. S8). As an additional check for unintended mutations, we called variants in regions predicted to be off-target gRNA-binding sites and found that no SNPs or indels existed in the exons near these regions (*SI Appendix*, Text 7 and Table S4)*.*

### Feeding Assay with GSL Supplemented *A. thaliana* Quad-GSL and *P.* *brassicae* Mutants.

We grew *A. thaliana* quad-GSL mutants for GSL supplemented feeding assays under short day conditions (8D:16L) at 25 °C. Following the procedures outlined in Schramm et al. (38), rosette leaves were cut from 7-wk-old plants at petioles and placed into either one of three aqueous GSL treatments (50 µl of 20 mM sinigrin, benzyl-GSL, or I3M-GSL) or in water as a negative control. Neonate *P. brassicae* larvae from mutant and control lineages were then introduced to the treated leaves (N = 10 per treatment, per lineage). Petri dishes were prepared so that 10 larvae from the same lineage fed on two leaves from the same GSL treatment group. Larvae fed on the plants under long day conditions (16L:8D at 25 °C). Leaves were changed every 24 h to maintain the level of intact GSLs in the leaves. After 5-d feeding, we measured larval weight individually. To assess the amount of intact GSLs in the experimental leaves, we also prepared three additional leaves for each treatment and freeze-dried them after 24-h incubation at 25 °C. The amount of intact GSLs in the leaves were analyzed by high performance liquid chromatography with UV Detector (HPLC–UV) as described in *SI Appendix*, Text 8*.*

### Feeding Assay with Brassicales Host Plants and *P. brassicae* Mutants.

We prepared the host plants *B. juncea*, *B. oleracea*, *T. majus*, and *A. thaliana* (quad-GSL: *myb28myb29cyp79B2cyp79B3* line). *B. juncea*, *B. oleracea*, and *T. majus* were grown under long-day conditions (16L:8D at 25 °C), and *A. thaliana* was grown under short-day conditions (8D:16L at 25 °C). For each plant species included in the assay, three 7-wk-old plants were placed in a mesh cage under long-day conditions. Twenty neonate (approx. 12 h old) *P. brassicae* larvae from one of four lineages (WT, *ΔNSP*, Δ*MA*, and Δ*NSP*Δ*MA*) were placed on plant leaves with a soft paintbrush. In total, 80 neonates per *P. brassicae* lineage were split across the four plant species. After 5-d-feeding, the weight of each larva was measured. To assess the GSL profile differences of each plant species, we analyzed GSLs in the leaves using the desulfo-GSL method (57) (*SI Appendix*, Text 8).

To assess if batch effects from our rearing conditions had any effect on our results, we repeated this feeding assay at a later date, using *B. juncea*, *B. oleracea*, and GLS-null *A. thaliana*. Larval growth rates in this repeated experiment confirmed many of the patterns seen in our main results, with Δ*NSP* lines having decreased performance on *B. juncea*, Δ*MA* lines having decreased performance on *B. oleracea,* and Δ*NSP*Δ*MA* lines performing poorly on everything but GSL-null *A. thaliana* (*SI Appendix*, Text 2 and Fig. S7).

### Estimating Selection Strength for Three *Pieris* Species.

To identify single-copy orthologous genes in three *Pieris* species (*P. napi, P. rapae*, and *P. brassicae*), we used the tool SonicParanoid v. 1.3.4 (58) on protein sets generated from reference genomes (*SI Appendix*, Text 12 and Table S7). A total of 8,695 single-copy orthologs (SCO) groups were identified. Using a published Pool-seq dataset (*P. napi*) (59), and two generated for this study (*P. brassicae, P. rapae;* *SI Appendix*, Text 13) (60), each containing 24 individuals, reads were filtered and cleaned with SAMtools (61), and then mapped to their respective reference genomes with NextGenMap (v 0.5.5) (62). We next generated an mpileup file and extracted 20 sequences per species for each SCO with the sampling-pileup2fasta-mauanno-NEW.pl script, for a total of 60 sequences per the SCO group. Only reads with MapQ scores > 20 and less than 10% missing data were included in our analysis, which reduced our dataset from 8,695 SCO groups to 4,790 groups. Because *NSP* has two tandem copies in *P. brassicae* and *P. napi* and therefore does not have a MapQ score > 20 (due to reads mapping to both copies), we used an alternative approach. First, we removed the MapQ score filter and extracted reads mapping to the *NSP* locus. Then, for species with *NSP* duplicates, we extracted 10 reads from each *NSP* copy and placed them into one input file for *NSP* per species. This combined input will underestimate the selection index of *NSP* rather than overestimating it, as it introduces more polymorphism within the gene because there are no fixed differences between loci (*SI Appendix*, Table S7). Detection of positive selection in the combined NSP input should thus be considered strongly supported.

Extracted reads for each SCO group were used as input for the HDMKPRF software (48) to identify genes under strong selection in each species. Following author recommendations, we ran 200,000 burn-in steps followed by 300,000 steps of the Markov chain Monte Carlo (MCMC) process to produce posterior distributions of the parameters (-bs 200000 - ts 300000 flags). The species relationship we used as input was (P. napi (P. rapae, P. brassicae)). Output from this software was plotted in R (53), using the tidyverse package (63). All HDMKPRF tests were run in triplicate and assessed for convergence via comparison of the estimated selection coefficients (*SI Appendix*, Fig. S9). As in Zhao et al. (48), we considered a gene to be under positive selection in its lineage when more than 97.5% of its MCMC sample points had selection intensities > 0, with higher values suggesting stronger positive selection relative to other genes. Full output tables from each HDMKPRF run, as well as results for classic MK tests (49) for each gene can be found in Dataset S1. A full set of results is reported in *SI Appendix*, Table S8. Per-gene estimates of nucleotide diversity (π) and Tajima’s *D* for all single-copy orthologous and NSP-family genes shared between our three chosen *Pieris* species were calculated with the software PoPoolation v. 1.2.2 (52) and are reported in Dataset S1.

## Supplementary Material

Appendix 01 (PDF)

Dataset S01 (XLSX)

## Data Availability

[Genome assembly] data have been deposited in [ENA] (PRJEB51614) (64). Pool-seq reads used for selection analyses can be found on the NCBI Sequence Read Archive under BioProject PRJNA832077.
