# Supplementary material for: Testing hypotheses of a coevolutionary key innovation reveals a complex suite of traits involved in defusing the mustard oil bomb
Source: Proc Natl Acad Sci U S A. 2022 Dec 12;119(51):e2208447119. doi: 10.1073/pnas.2208447119 (PMC9907077; doi:10.1073/pnas.2208447119)
Supplement: Supplementary file 1 — Appendix 01 (PDF) [file pnas.2208447119.sapp.pdf]

PNAS

Supplementary Information for
Testing hypotheses of a coevolutionary key innovation reveals a complex suite of traits involved in defusing the mustard oil bomb

Authors: Yu Okamura[1,2,3*], Hanna Dort[4], Michael Reichelt[5], Christopher W. Wheat[4], Heiko Vogel[1,2]

Affiliations:
(1) Department of Entomology, Max Planck Institute for Chemical Ecology, Hans-Knöll-Str. 8, Jena, Germany 07745
(2) Department of Insect Symbiosis, Max Planck Institute for Chemical Ecology, Hans-Knöll-Str. 8, Jena, Germany 07745
(3) Department of Biological Sciences, Graduate School of Science, University of Tokyo, Tokyo, Japan
(4) Department of Zoology, Stockholm University, Stockholm, Sweden SE-10691
(5) Department of Biochemistry, Max Planck Institute for Chemical Ecology, Hans-Knöll-Str. 8, Jena, Germany 07745

*To whom correspondence should be addressed.
Email: yokamura@ice.mpg.de

This PDF file includes:

Supplementary Text 1-16
Figures S1 to S9
Tables S1 to S8
SI References

Supplementary Results

Text 1. *In vitro* NSP/MA activity assays and fecal analyses confirmed the loss of GSL detoxification function in double-KO, CRISPR/Cas9 modified larval lines.

To assess the role of NSP and MA detoxification performance on larval fitness, we used the CRISPR/Cas9 technique to create three knockout (KO) lines of *P. brassicae*: a homozygous Δ*NSP* line, a homozygous Δ*MA* line, and a Δ*NSP*Δ*MA* line lacking both *NSP* and *MA* genes (see *SI Appendix*, Fig. S2, S3). To quantify the loss in GSL detoxification ability within each of our three mutant *P. brassicae* lines, we extracted gut proteins from all KO lineages, as well as a wild-type control lineage, and checked their activity against different GSLs with an *in vitro* assay. Extracted proteins were mixed with one of four selected GSLs, that are common among their host plants (*SI Appendix*, Fig. S6), and then exposed to myrosinase, which hydrolyzes GSLs to create toxic ITCs. In wild-type samples, NSPs worked as expected, with GSLs overwhelmingly converted to nitriles instead of ITCs (*SI Appendix*, Fig. S5). Single-KO samples (i.e. Δ*NSP* and Δ*MA* lines) were somewhat less effective at this conversion, resulting in detectable ITC accumulation. Strikingly, Δ*NSP*Δ*MA* samples showed no evidence for conversion of ITCs to nitriles, indicating that the ability to detoxify GSL compounds was completely lost in Δ*NSP*Δ*MA* larvae (*SI Appendix*, Fig. S5).

Although the above *in vitro* assay results demonstrated remarkable activity differences between wild type and Δ*NSP*Δ*MA* line larval gut samples, the activity differences between the Δ*NSP* and the Δ*MA* lines were difficult to detect. Thus, we made a dilution series of total gut-extracted protein and repeated our activity assay to compare the functional efficiency of each sample against different GSLs. We found that wild-type samples showed the highest efficiency of nitrile forming activity and Δ*NSP*Δ*MA* samples did not increase the amount of detected nitriles, even with higher gut-protein concentration (Fig. 3A, main text). There were differences in functional efficiency between Δ*NSP* and Δ*MA* lines against I3M and 4MSOB GSLs, potentially highlighting the functional differences between NSP and MA. In other words, NSP and MA may indeed have different efficiencies against different GSL compounds.

In a complementary approach to the gut assays, we analyzed feces from *P. brassicae* mutant lines to assess how the absence of NSPs affected *in vivo* GSL breakdown (*SI Appendix*, Text 11). Here, we detected nitriles derived from 4MSOB and ITCs derived from 4MSOB and 3MSOP. Furthermore, we found more than 100x accumulation of ITCs in the feces from Δ*NSP*Δ*MA* individuals, demonstrating that the Δ*NSP*Δ*MA* line completely lost its ability to convert GSLs to nitriles (*SI Appendix*, Fig. S5).

Text 2. Replication of the feeding assay with wild Brassicaceae plants.

Although we found dramatic growth level differences between wild type and Δ*NSP*Δ*MA* in the feeding assay using wild Brassicaceae plants, our experimental design had potential problems with batch effects. Therefore, to confirm the pattern we found in this feeding assay, we performed the same experiments using *Brassica juncea*, *Brassica oleracea*, and *Arabidopsis thaliana* (quad-GSL: *myb28myb29cyp79B2cyp79B3* line) again. This new assay, lacked *Tropaeolum majus* due to time constraints/the plant being unavailable at the time. The results are shown in (SI Appendix, Fig. S7). The consensus trends between the two replications were (1) Δ*NSP* grew worse compared to WT or Δ*MA* and Δ*NSP*Δ*MA* could not grow on *B. juncea*. (2) Δ*MA* and Δ*NSP*Δ*MA* grew worse compared to WT or Δ*NSP* on *B. oleracea*. On GSL-null *Arabidopsis*, overall growth levels among the KO lines were at a similar level.

Supplementary Experimental Procedures

Text 3. gDNA extraction and genome sequencing

We extracted gDNA from a single *P. brassicae* pupa from the laboratory population of Wageningen University (Netherlands). The pupa was frozen with liquid nitrogen and stored at -20°C until gDNA extraction. We used a Nanobind Tissue Big DNA Kit (circulomics) for extracting gDNA. For MinION sequencing, the extracted gDNA was size selected with a Short Read Eliminator XS kit (circulomics) before library preparation. We used 1.5μg of size-selected gDNA for MinION library preparation. We used a Ligation Sequencing Kit (SQK-LSK109) to generate a library and sequenced it with a FLO-MIN106D Flowcell (R 9.4.1). For additional Illumina short-read sequences, we used gDNA from the same individual without size selection. We sequenced this gDNA with Illumina Hiseq 2500 (150bp paired-end).

Text 4. Genome assembly and annotation

We used guppy basecaller ver. 4.0.11 (1) to perform high-accuracy base calling with dna_r9.4.1_450bps_hac.cfg models. We obtained 19.1 GB of reads with an N50 size of 7.8KB. The called bases were assembled with Flye 2.7 (2) and NECAT (3) separately. For the Flye assembly, we set the estimated genome size to 300mb and used the --nano-raw option. To recover sequences with biased coverage, such as mitogenomes, we also added the --meta option for the assembly. For our NECAT assembly we used the default options. The two genomes generated from different assemblers were separately polished with Racon(4) for four rounds 1.4.13, using (-m 8 -x -6 -g -8 -w 500) settings. The Racon polished genomes were additionally polished using Medaka 1.0.3 (https://nanoporetech.github.io/medaka) with the r941_min_high_g344 model. To get haplotype genomes, we ran PURGEhaplotigs 1.0.3 (5) with the default settings. The generated two polished haplotype genomes were merged with quickmerge v0.3 (6). The merged genome was polished with Illumina short reads using ntEdit v1.3.2 (7). We ran BUSCO ver. 4 (8, 9) with the lepidoptera_odb10 database (10) to assess the completeness of the generated final genome (*SI Appendix*, Table S2).

We masked the repeat sequences in the genome to reduce the complexity for gene prediction. We used RepeatModeler ver. 1.0.7 (11), which implemented RECON ver. 1.08 (12) and RepeatScout ver. 1.0.5(13), and also used Tandem Repeats Finder (TRF) (14) to predict the repeat structure. We mapped available RNAseq data (Shore Read Archive accessions: ERX2829498-ERX2829499) of *P. brassicae* to the masked genome with STAR 2.7 (15) and used it as a hint for subsequent genome annotation. We softmasked the genome with BEDTools ver. 2.27.1 (16) and used this genome for the BRAKER2 genome annotation pipeline (16) with the --softmasked option. The acquired predicted gene sequences were isoform filtered with AGAT (17) and then analyzed with BUSCO ver. 4 (8, 9) to assess the completeness of the annotation with the lepidoptera_odb10 database (10). Overall, 16, 335 genes were annotated, and 96.7% of BUSCO genes were complete and existed in single copy (*SI Appendix*, Table S2). The raw data and genome assembly are under ENA accession PRJEB51614.

Text 5. RNA extraction prior to qPCR

We used the innuPREP RNA Mini Kit 2.0 (Analytik Jena) for RNA extraction. We designed primers of *NSP*, *MA*, and *SDMA* for RT-qPCR using Primer3Plus with setting product size = 70-180bp, Tm = 59-61°C, GC% = 40-60% and Max Poly-base = 3 (*SI Appendix*, Table S3). We also designed primers for *EF1α* and used it as a reference gene. We used PrimeScript™ RT Master Mix with gDNA Eraser for cDNA synthesis. We ran RT-qPCR reactions with a CFX Connect Real-Time PCR Detection System (BIO-RAD) using TB Green® Premix Ex Taq™ II (Tli RNaseH Plus) with two technical replicates and three biological replicates for each sample. We checked specific amplification by performing a melting curve analysis from 65°C to 95 °C and also confirmed no gDNA contamination using RNA samples without cDNA synthesis. We calculated relative gene expression levels by the ddCT method normalized by *EF1α* (47).

Text 6. Generating sgRNAs for CRISPR/Cas9 injections

Candidate sgRNA target sites were identified for each gene through searches with ZiFiT Targeter Version 4.2 (18, 19)(SI Appendix, Table S3). To get per-gene SNP distributions, each target gene was cloned and Sanger sequenced from four *P. brassicae* individuals from our lab population (Wageningen University, NL). We decided on sgRNA target sites that did not include SNPs. Off-target effects were assessed with the cas9off tool (20, 21)(https://github.com/wangqinhu/cas9off).

Text 7. Genotyping and hand-pairing G0 KO lines, and assessing off target effects in the fixed KO lines

We numbered each G0 *P. brassicae* adult and we used one of the middle legs of each adult for gDNA extraction and genotyping. We extracted gDNA with the 10 % Chelex 100 resin in water with subsequent homogenization and performed PCR with gene-specific primers to amplify the region where the gRNA target sites were located (SI Appendix, Table S3). Since there are two *NSP* copies in our wild type line of *P. brassicae*, we selectively amplified each *NSP* copy, cloned, and sequenced them to confirm the genotype (SI Appendix, Table S3). After Sanger sequencing, we selectively paired individuals with frameshift-mutations by hand pairing and acquired G1. We took the same rearing and genotyping methods and generated homozygous mutant lines for *NSP* and *MA*. The generated homozygous *NSP* knockout line (Δ*NSP*) had a 5 nucleotide (nt) deletion in the first exon of both *NSP* gene copies, and the homozygous *MA* knockout line (Δ*MA*) had two mutations (1nt insertion or 1 nt deletion) in exon 1 (Fig. S3). A double-KO line (Δ*NSP*Δ*MA*) was generated by injecting *MA* gRNA into eggs from the Δ*NSP* line; this had one 5nt deletion in the first exon of both *NSP* copies and two mutation variants in *MA* (1nt insertion or 13nt insertion in exon 1). Since both of the mutations observed in our homozygous mutant lines disrupt some restriction enzyme recognition sites, we also used PCR-RFLP for genotyping our KO lines. The mutation at *NSP* in both Δ*NSP* and Δ*NSP*Δ*MA* lines disrupted the recognition site of *Sty*I, and that of Δ*MA* disrupted the recognition site of *Eco*R1. We designed primers for PCR-RFLP (SI Appendix, Table S3) and digested the PCR fragments with those restriction enzymes to further confirm our genotyping by sequencing.

To assess the potential off-target effects in KO lines, we sequenced the whole genome of generated KOs (Δ*NSP*, Δ*MA*, and Δ*NSP*Δ*MA*) with MinION following the same protocol described above in SI Appendix, Text 3. Acquired reads were mapped to the wildtype genome with minimap2(22, 23), and SNPs were called by Clair3 v0.1-r11(24)(https://github.com/HKU-BAL/Clair3) using a bed file including position information for where gRNAs could potentially bind based on cas9off results. The results are shown in SI Appendix, Table S5. In short, there were no SNPs located in coding regions that were found near (within 10bp of) the potential gRNA binding sites in any KO line.

Text 8. Quantification of host plant GSL content

Prior to our feeding assays, we collected leaves from three individuals per species per GLS-null *A. thaliana* treatment group and then froze them with liquid nitrogen. These samples were then freeze-dried and ground by metal beads in a shaker. We used 10mg of ground leaf material for the chemical analysis. We added 80% methanol with adequate internal standard GSLs for extraction. We used benzyl GSL for *Brassicaceae* species, sinigrin for *T. majus* and sinalbin for *A. thaliana*, as an internal standard. The extraction and analysis by HPLC-UV followed the method described in Burow et.al. (32). We incubated samples for 5 minutes with 230 rpm of shaking, then centrifuged the samples at 130,000 rpm for 10 minutes. We took the supernatants to DEAE sephadex A-25 columns and added 1 ml of MES buffer pH5.2 and 30 μl of arylsulfatase solution (Sigma-Aldrich) after washing with 80% methanol and 2ml of water. We incubated the column overnight at room temperature and eluted each column with 0.5ml water. The samples were analyzed using HPLC-UV (Agilent 1100 HPLC system, Agilent Technologies) with a reverse-phase C-18 column

(Nucleodur Sphinx RP, 250 mm × 4.6 mm, 5 µm, Machrey-Nagel, Düren, Germany). We identified the desulfo GSLs based on the retention time and UV spectra with known standards. Detection was performed with a photodiode array detector and peaks were integrated at 229 nm. Chemical analyses, feeding assay, and qPCR data are stored under the DOI https://doi.org/10.17617/3.AMIAIE at EDMOND (https://edmond.mpdl.mpg.de/).

Text 9. Statistical analyses

In order to test whether mutant larvae differed in their growth depending on the treatment in our feeding assays (different host plants or spiked-in GSLs), the method of generalized least squares (gls, nlme library (25, 26)) with the varIdent variance structure was used. The appropriate variance structure (allowing a different variance for each treatment (plant species or spiked-in GSLs), each larval mutant, or each treatment-larval mutant combination) was determined by running different models with the three different variance structures and choosing the model with the smallest AIC. Finally the variance structure for each treatment-larval mutant combination was chosen. P-Values were obtained by removing successively the explanatory variables and comparison of models with and without an explanatory variable with likelihood ratio tests (27). In order to find out which treatments led to different growth of the larvae, and which larval mutant grew differently, factor level reduction was applied (28). To find out which treatment-larval mutant combinations grew differently, pairwise Welch two sample t-tests were applied in case of normally distributed data and Wilcoxon rank sum tests with continuity correction when data were not normally distributed. All obtained p-values were corrected to adjust for multiple comparisons using the false discovery rate. All analyses were done in R v. 4.2.0. (29) The results are shown in *SI Appendix*, Table S6.

Text 10. Gut activity assays

We dissected larvae from each of our four *P. brassicae* lines and extracted their midguts. We washed out the gut contents with MES buffer (50mM pH7.0) and immediately submerged the dissected gut in 4°C MES buffer (50mM pH7.0) with protease inhibitor (Halt™ Protease Inhibitor Cocktail, Thermo Scientific™). We homogenized each gut with metal beads for two minutes and then centrifuged them for two minutes at 10.000xg. We removed the supernatant and measured the total protein concentration with NanoPhotometer®. Each gut sample was diluted with a MES buffer to have three different concentrations (2µg/µl, 1µg/µl, 0.5 µg/µl) of total proteins in 50µl. We prepared four GSL solutions (Sinigrin, 4MSOB-GSL, Benzyl-GSL and I3M-GSL, Phytoplan) with 2mM in 50µl for each sample. We mixed 50µl of gut extract and 50µl of GSL solution and added 8ng of myrosinase (Sigma-Aldrich) to the mix to start the reaction. After 30 minutes of incubation at 25°C, we stopped the reaction by adding solvents. For samples with sinigrin and benzyl-GSL, we added 1ml of ethyl acetate with internal standard (benzonitrile) to stop the reaction. We vortexed and centrifuged the mix and used the ethyl acetate phase for GC-MS analysis. For samples with I3M-GSL and 4MSOB-GSL, we stopped the reaction by adding 400µl of methanol. The samples were filtered, and diluted samples were analyzed by LC-MS/MS.

GC-MS analysis was conducted using an Agilent 6890 Series gas chromatograph. Helium was used as the carrier gas and the outlet of the column (DB5MS 30m x 0.25mm x 0.25um film) was coupled to a Agilent 5973N quadrupole mass detector (Agilent Technologies, Waldbronn, Germany). Parameters for electron impact sample ionization were as follows: interface temperature, 270°C; repeller, 30V; emission, 34.6uA; electron energy, 70eV; source temperature, 230°C. Mass spectrometer was run in scan mode in the mass range m/z 33 to 300. The chromatographic conditions were: splitless injection at 220°C, we used two different chromatographic gradients for the analysis of the breakdown products of sinigrin and benzyl-GSL. For sinigrin: initial oven temperature, 35°C for 3 min, increased at 12°C/min to 200°C followed by an increase of 60°C/min to 270°C and hold for 3 min; for Benzyl-GSL and Phenylethyl-GSL: initial oven temperature, 45°C for 3 min, increased at 12°C/min to 175°C followed by an increase of 60°C/min to 270°C and hold for 2 min. The following breakdown products were quantified by the peak area of the respective extracted ion chromatogram: allyl-cyanide (aka 3-butenylnitrile) m/z 67; allyl isothiocyanate m/z 99; Benzylcyanide m/z 117; Benzyl isothiocyanate m/z 149; phenylethyl cyanide (aka

phenylpropylnitrile) m/z 131; phenylethyl isothiocyanate m/z 163; indole-3-acetonitrile (indol-ACN) m/z 155; internal standard benzonitrile (aka phenyl cyanide) m/z 103. Peak area of the analyte peaks were normalized to the peak area of the internal standard benzonitrile.

LC-MS/MS analysis was performed on an Agilent 1260 series HPLC system (Agilent Technologies) coupled to a tandem mass spectrometer QTRAP6500 (SCIEX, Darmstadt, Germany). Separation was achieved on a Zorbax Eclipse XDB-C18 column (50 × 4.6 mm, 1.8 mm; Agilent) with a solvent system of 0.05% formic acid (A) and acetonitrile (B) at a flow rate of 1.1 ml/min. The elution profile was the following: 0 to 0.5 min, 3% to 15% B; 0.5 to 2.5 min, 15% to 85% B in A; 2.5 to 2.6 min, 85% to100% B, 2.6 to 3.5 min, 100% B and 3.6 to 6.0 min 3% B. Electrospray ionization (ESI) in positive ionization mode was used for the coupling of LC to MS. The mass spectrometer parameters were set as follows: ion spray voltage, 5500 V; turbo gas temperature, 600 °C; collision gas, medium; curtain gas, 45 psi; ion source gas 1, 60 psi; ion source gas 2, 60 psi. Parent ion to product ion was monitored by multiple reaction monitoring (MRM) as follows: m/z 146 →129 (collision energy [CE], 13 V; declustering potential [DP], 20 V) for 4-methylsulfinylbutyl cyanide (4MSOB-cyanide, aka 5-methylsulfinylpentylnitrile); m/z 178 →114 (CE, 13 V; DP, 20 V) for 4-methylsulfinylbutyl isothiocyanates (4MSOB isothiocyanate); m/z 157 → 130 (CE, 15 V; DP, 20 V) for indole-3-acetonitrile (indol-ACN); m/z 130 → 77 (CE, 37 V; DP, 20 V; m/z 130 is the insource fragment of the compound) for indol-3-carbinol; m/z 132 →115 (CE 13 V; DP 20 V) for 3-methylsulfinylpropyl cyanide (3MSOP-cyanide, aka 4-methylsulfinylbutylnitrile); m/z 164 →100 (CE, 13 V; DP, 20 V) for 3-methylsulfinylpropyl isothiocyanate (3MSOP isothiocyanate). Data processing was performed using the software Analyst v.1.5 (Applied Biosystems).

The acquired peak areas from GC-MS and from LC-MS/MS were compared among samples to assess the amounts of nitriles and/or ITCs that were formed. To make an activity curve along with the dilution series of each sample, we first confirmed that not all the GSLs were converted to nitriles in the samples with the highest amount of gut protein. Then, we made a linear regression curve based on nitrile or ITC peak area data and compared the slopes among *P. brassicae* lines. (Figure 3A, main text). We tested for significant differences between the slopes with an ANOVA in R (29).

Text 11. GSL breakdown product analysis in the feces of KO lines

We prepared five final instar larvae from each KO line. These were reared on *B. oleracea* and we separately put each larva in a petri dish with *B. oleracea* leaves. We collected fresh feces every 30 minutes and stored them at -20°C. We did this collection for eight rounds. Collected feces were weighed and feces content was extracted with methanol. We used 500µl of methanol for 200mg of feces. The feces extracts were analyzed by LC-MS/MS as described in Text 10 and the relative amount (peak area per mg weight) of the GSL breakdown products of 3MSOP-GSL: 3-methylsulfinylpropyl cyanide (3MSOP-cyanide), 3-methylsulfinylpropyl isothiocyanate (3MSOP isothiocyanate), of 4MSOB-GSL: 4-methylsulfinylbutyl cyanide (4MSOB-cyanide), 4-methylsulfinylbutyl isothiocyanates (4MSOB isothiocyanate), and of I3M-GSL: indol-3-acetonitrile (indol-ACN), and indol-3-carbinol were compared among samples.

Text 12. Generating protein sets for *Pieris* species.

Protein sets for each of three *Pieris* species (*P. napi, P. rapae*, and *P. brassicae*) were generated from reference genomes using genome annotations that were filtered with the AGAT (v.0.8.0) agat_sp_keep_longest_isoform.pl script (17) to include only the longest isoform per gene. To this end, we used the annotation for *P. brassicae* described in *SI Appendix*, Text 4. We generated *de novo* annotations for the Darwin Tree of Life (https://www.darwintreeoflife.org/) *P. napi* reference genome, as well as for the *P. rapae* reference genome (30), with the BRAKER2 automated annotation pipeline and included the –softmasked option (31). The Arthropoda reference protein set from OrthoDB v. 10 (10) was used as our training dataset for gene model prediction. Prior to annotation, reference genomes were softmasked with the redmask.py wrapper (v 0.0.2), which calls the tool RED (v. 05/22/2015) (32).

Text 13: Justification for use of HDMKPRF test, versus other tests of selection

Why did we choose the HDMKPRF test over a classic MK test or MKPRF test?
When deciding how to best estimate selection dynamics on *NSP* and *MA* at the species-level, we chose the HDMKPRF test (33) because it is a substantial advance on the more than 30-year-old MK test (34). The classic MK test was designed in the pre-genomics era, analyzing each gene locus separately. As a result, it has very low power to detect loci under weak or moderate selection (33). Moreover, the standard test statistics for the classic MK test cannot infer the directionality or relative strength of selection acting upon different genes being tested, making it difficult to compare one gene to another.

A major advance on the classic MK test was made with the creation of the MKPRF test, which uses the full set of analyzed genes to estimate changes in effective population size and mutation rate as part of its Bayesian analysis (35, 36). The MKPRF test ultimately estimates selection intensity (rather than a selection coefficient) per locus (35, 36). However, it is unable to polarize changes to specific lineages or branches (i.e. in an analysis of two species, it cannot identify on which species branch selection has occurred). The ability to polarize results is the substantial advance of the HDMKPRF over the MKPRF approach, allowing us to analyze multiple species and 1000s of genes within one modeling framework (33). This allows for the estimation of demographic, mutation and selection dynamics, with the former two informing on the latter, providing substantially greater statistical power than the classical MK test.

Why didn't we choose to do branch or branch-site tests?
Branch tests for *NSP* and *MA* have already been conducted in previous studies, finding that some of the *NSP*-genes have higher dN/dS ratios than genome wide averages along branches consistent with hostplant shifts (37). However, some of the calculated dN/dS values were < 1(37), meaning that these results, while significant, could be due to either relaxed selection or positive selection. Other studies with different taxa have found evidence for *NSP* branches having dN/dS > 1 (38). Branch site tests have also been conducted for our genes of interest, finding a codon in *NSP* that was under positive selection (37).

Perhaps more importantly, branch and branch site tests only detect positive selection under a very narrow set of evolutionary scenarios, using the divergence between species while ignoring variation within them (39). Moreover, they are misleading when using data from individuals within a species (40). Since such analyses use a single consensus sequence per species, in the case of our study, they would rely upon a very limited phylogenetic tree (with only three species). The result would be an extremely underpowered analysis, as dN/dS methods derive appropriate statistical power when upwards of a dozen taxa are used to detect selection, with four species being the absolute minimum recommended for analysis (41). With these considerations in mind, we did not think branch or branch-site tests to be appropriate for our dataset. Additionally, we wished to gain more power via using extensive polymophsm data within species.

Is detecting 30% of genes as being under selection reasonable? Or is the output of the HDMKPRF test inflated?

We realize that the number of genes we detected as being under positive selection are a lot higher than what one might expect with a classic MK test, but this is to be expected because, as discussed earlier in this section, classic MK tests often do poorly at detecting weak or moderately strong selection. When comparing the results of tests of selection, both the MKPRF and HDMKPRF both detect nearly 10 times more genes under positive selection than standard MK tests (33, 35, 36). While this sounds like a lot, it is not. Rather, it is directly concordant with estimates of alpha, the proportion of nonsynonymous substitutions fixed by positive selection (42). This estimate is upwards of 0.54 for other insects with large population sizes (42, 43), meaning that we can expect ~50% of genes to exhibit some degree of positive selection. With

this in mind, our finding of approximately 30% of genes having experienced positive selection within each lineage is not that extreme. However, we would like to note that MKPRF-based tests do have their drawbacks, and have the potential to incorrectly identify linked neutral loci as being under positive selection. For a discussion of these issues, see Li and Costello (44). Additionally, the authors of the HDMKPRF highlight how the test likely underestimates negative selection, but this is not a focus of our paper's analysis.

Text 14: Calculating pi and Tajima's *D*

To estimate nucleotide diversity (π) and corrected Tajima's *D* values for the coding regions of every gene in each of three *Pieris* species, we used the scripts included in Popoolation v.1.2.2 (45). For estimations of π, our inputs were an exon-only annotation file and a pileup file created from our poolseq reads with no MapQ filter. Estimations were made with the variance-at-position.pl script with the options --measure pi --pool-size 48 --min-count 2 --min-coverage 20 --snp-output --max-coverage 150 --fastq-type sanger.

For calculations of corrected Tajima's *D* values, we used subsampled versions of the pileup files described above. These subsampled files contained exactly 20 reads per position and were created with the PoPoolation "subsample-pileup.pl" script (45). Regions with lower than 20x coverage or higher than 200x coverage were excluded from the dataset. We estimated Tajima's *D* values with the variance-at-position.pl script. Here, we used the options --measure D --pool-size 48 --min-count 2 --min-coverage 4 --min-covered-fraction 0.4 --snp-output --max-coverage 500 --fastq-type sanger.

Text 15. Generating pool-seq data for tests of selection

Pool-seq data for *P. brassicae* and *P. rapae* populations were gathered following the methods of Keehnen *et al.* 2018 (46). Briefly, 24 adult individuals per species were collected from single populations, and DNA was extracted from thorax tissue. High-quality DNA samples were then pooled at equimolar levels to 5 µg total and sequenced with paired-end, 100 bp long reads. *P. brassicae* individuals were sampled from a population in 2014 near El Brull, Spain, and *P. rapae* individuals were sampled from a population in 2014 near the Cal Tet reservoir in the Delta del Llobregat Nature Area, which is located near Barcelona, Spain. These datasets were deposited in the SRA BioProject ID PRJNA832077. The pool-seq data used for *P. napi* was generated for a previous study, following the procedures above (46)(NCBI SRA Accession: SRX3901628). The pileup2fasta-mauanno-NEW.pl script was provided by Viola Nolte from her scripts for building the dataset for the MK analysis (47).

Text 16. Conflicting results and problems with heterologous expression of NSP/MA protein.

In Edger *et al.* (2015) (38), *in vitro* activity of NSP and MA was tested using proteins heterologously expressed in Sf9 cells. The activity of the obtained proteins was tested against two different GLSs, and nitrile-forming activity was found only for NSP but not for MA, leading to the conclusion that MA was inactive against the tested GLSs. In the current study using a gene editing approach, however, we found that (*in vivo*) MA also has nitrile-forming activity, which is in contradiction to previous findings using heterologously expressed MA protein. Based on numerous attempts to heterologously express functional NSP/MA proteins from different butterfly species in the subclade Pierini, we have found that even obtaining usable amounts of protein does not guarantee that the protein is functional in *in vitro* assays. For instance, in the Edger et al. (2015) paper (38), the authors tested the activity of not only the heterologously expressed NSP but also gut-extracted proteins. Although nitrile-forming activity was detected for the heterologously expressed NSP (NSP recombinant protein), the activity was far lower than that of gut-extracted protein. Additionally, of the two proteins (gut-extracted vs. heterologously expressed NSP), only the heterologously expressed NSP resulted in the formation of ITCs. These findings can in retrospect be seen as indicative of the technical difficulties arising from assays using heterologously expressed NSP or MA proteins. The absence of function, or lower level of function, in such assays could always be

due to complications of heterologous expression. Based on the above and numerous subsequent attempts in different expression systems, we conclude that (i) bacterial and yeast expression systems do not seem to be suitable for the production of functional NSP/MA proteins, (ii) functional NSP/MA proteins can be produced in insect cell line expression systems, but the amounts of NSP/MA protein required for most assays cannot be obtained easily, and (iii) yet unknown cofactors might be required for reliable, reproducible NSP/MA activity.

In sum, we conclude that the absence of nitrile-forming activity in previous heterologous expression studies, such as reported by Edger et al. 2015 (38), was due to technical limitations. In our present work, we have overcome these technical limitations by knocking out NSP/MA proteins by mutation of the coding gene, followed by performing activity assays with gut-expressed proteins *in vivo*. We conclude that our new, CRISPR/Cas9 NHEJ approach has avoided any uncertainties related to the difficulties in heterologous expression of our candidate proteins and has allowed us to unambiguously identify the nitrile-forming activity of MA.

Text 17. Data availability

While data availability is listed in each relevant subsection above, here we also gather these accessions here.

Genomics: The raw data and genome assembly for WT *P. brassicae*, as well as KO whole geome sequce data are under ENA accession PRJEB51614. Whole genome resequencing datasets for PoolSeq analyses are round under SRA BioProject ID PRJNA832077 (*P. rapae, P. brassicae)*, while *P. napi* is already published (SRA Accession: SRX3901628).

Biochemical assays, feeding assay results, and qPCR data: Chemical analyses, feeding assay, and qPCR data are stored under the DOI https://doi.org/10.17617/3.AMIAIE at EDMOND (https://edmond.mpdl.mpg.de/).

HDMKPRF results: Full tables of the HDMKPRF results, showing, e.g., the calculated selection intensities, DN, DS, PN, and PS for each SCO gene can be found in Excel file titled SI Data_File1. This file also includes traditional MK test results for each gene (Fisher's exact p-values).

Supplementary Figures

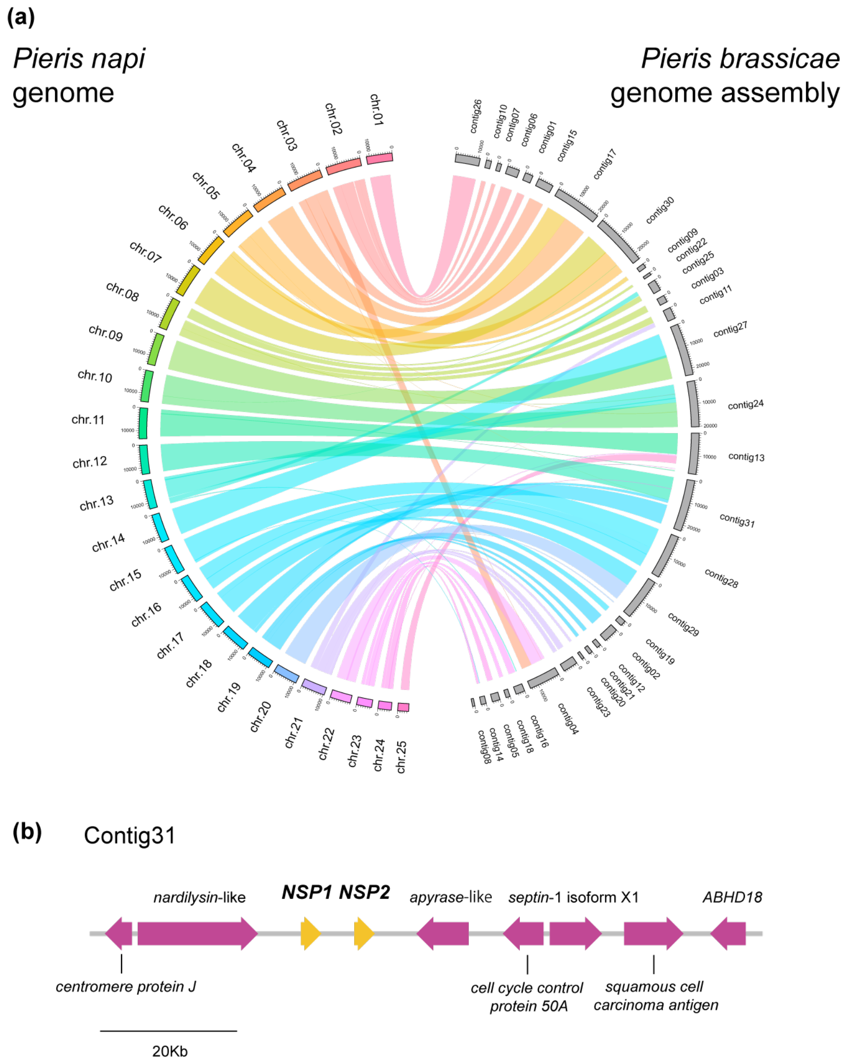

Fig. S1 (A) The genomic architecture of our generated *P. brassicae* genome compared to that of the published *P. napi* reference genome (25) (B) A depiction of the two tandem *NSP* genes found in the *P. brassicae* genome and the genes in their flanking region.

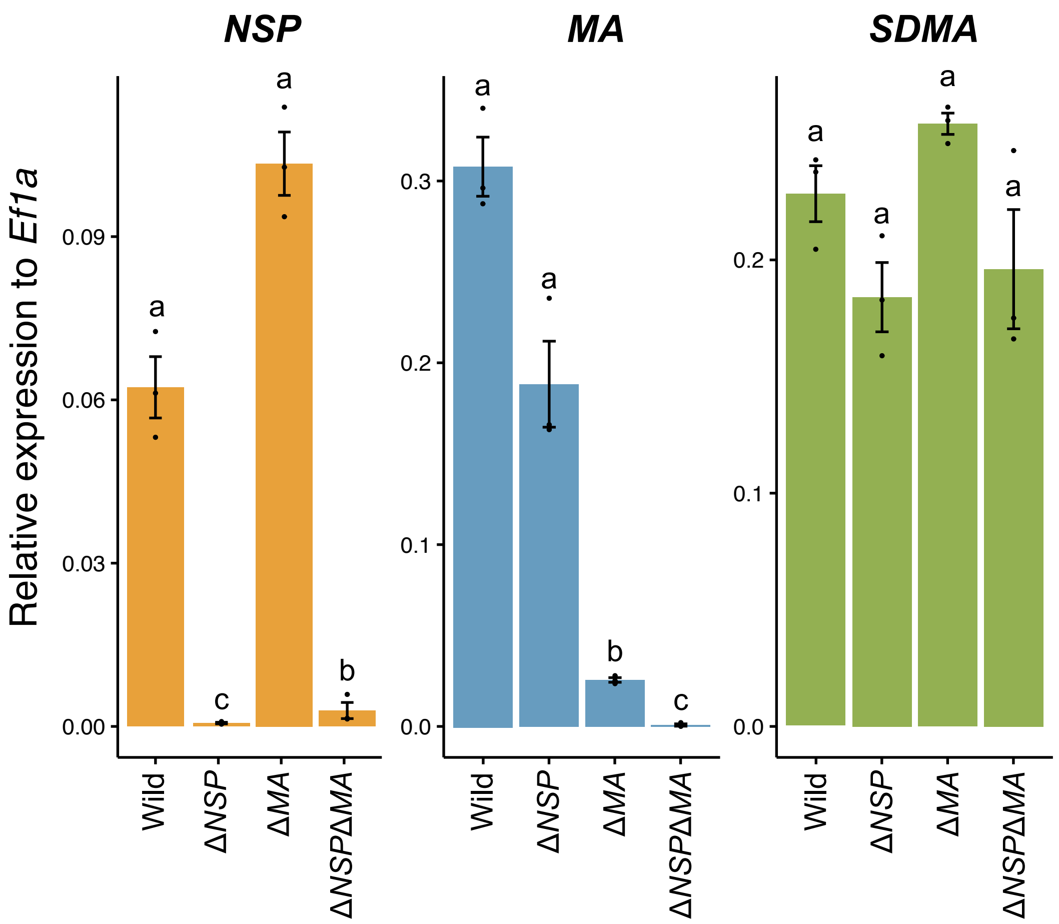

Fig. S2. qPCR analyses of *NSP*-family gene expression in each of three *P. brassicae* KO lines generated in this study, as well as for a wild-type line of *P. brassicae.* Different letters on each box show significance (pairwise t test on log-transformed expression values, FDR corrected p ≤ 0.05)

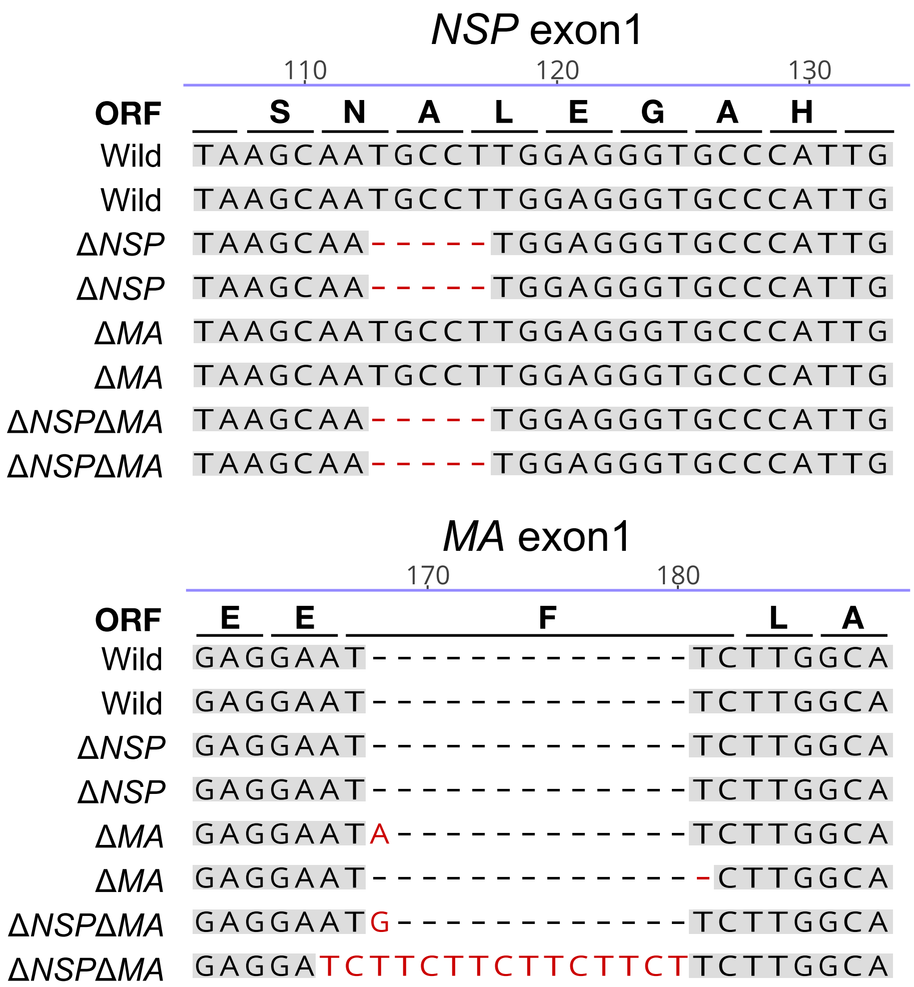

Fig. S3. Mutations caused by CRISPR-Cas9 mediating NHEJ in *NSP* and *MA*

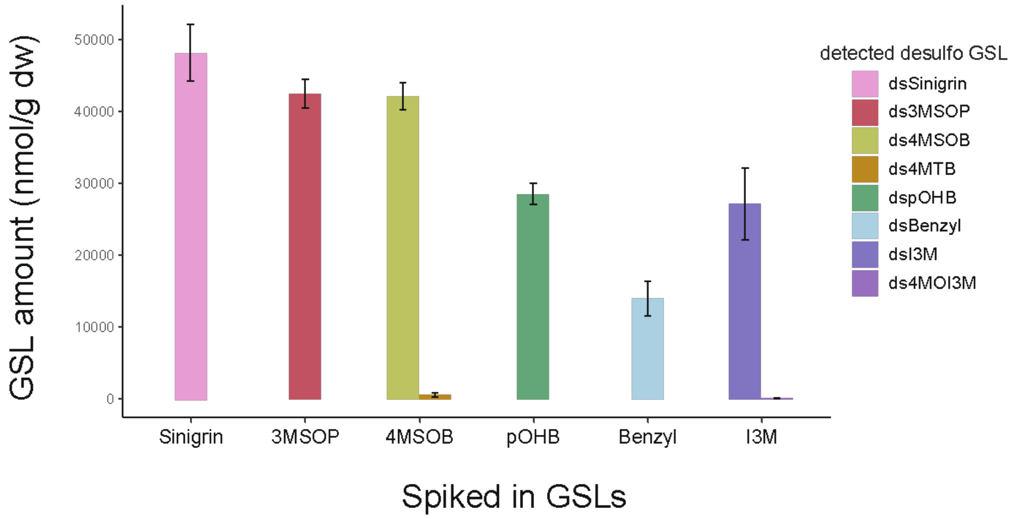

Fig. S4. GSL profiles of GLS-null *A. thaliana* plants with spiked in GSLs

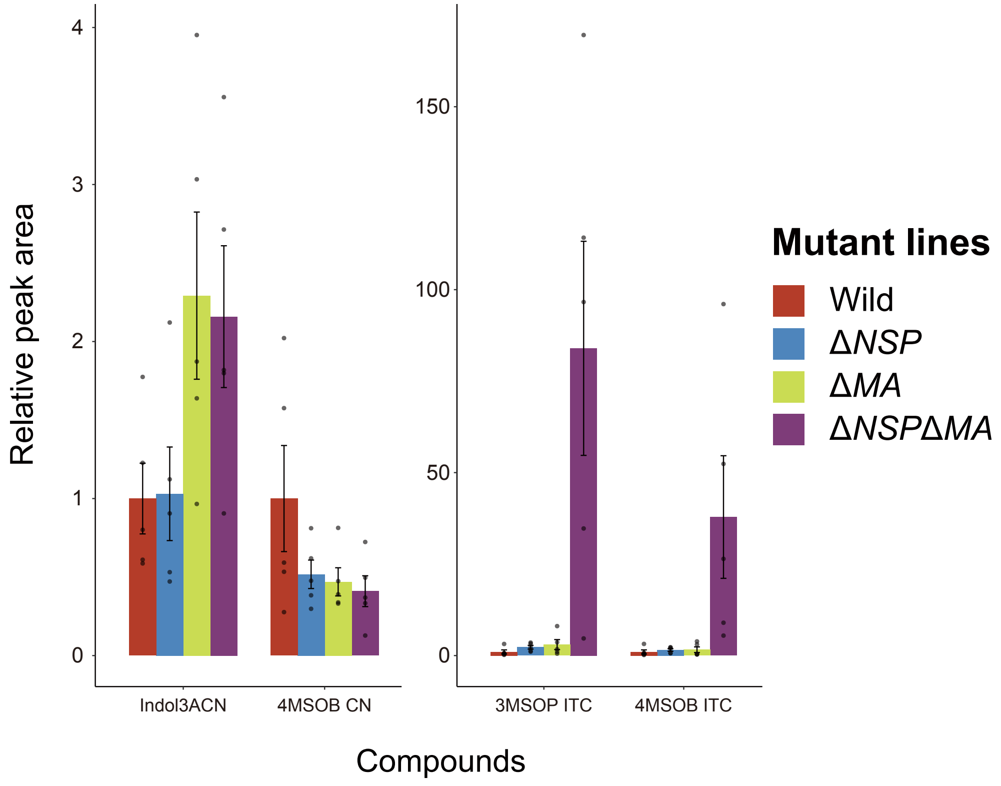

Fig. S5. GSL breakdown products detected in the feces of *P. brassicae* KO lines

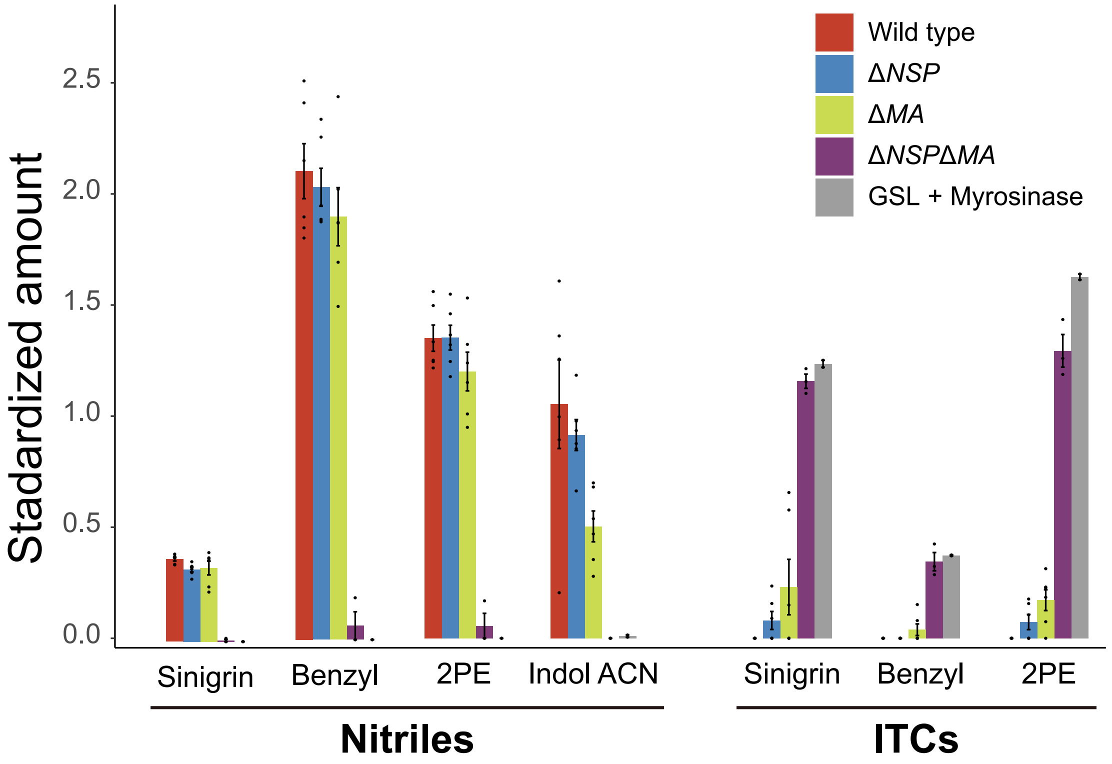

Fig. S6. Results from the gut activity assay at 1x gut protein concentration**.** Detected amounts of glucosinolate breakdown products are reported here for gut extracts from each line of *P. brassicae* larvae, as well as a negative control (GSL + myrosinase). Values here were standardized with the internal standard benzonitrile. ITCs: isothiocyanates. 2PE: 2‐phenylethyl glucosinolate, Indol ACN: Indole-3-acetonitrile.

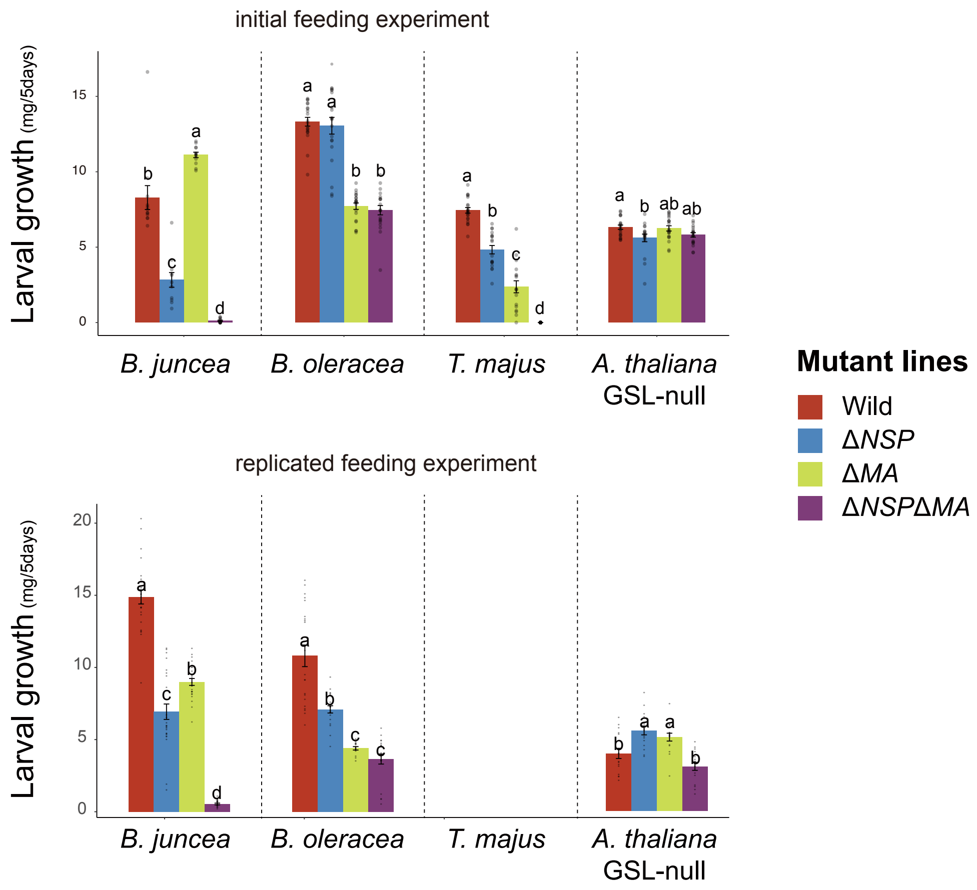

Fig. S7. Results of two replicated feeding assays using wild Brassicaceae plants. *T. majus* was not included in the replicated experiment. The consensus trends on *B. juncea* were that Δ*NSP*Δ*MA* individuals did not grow and that Δ*NSP* individuals grew worse compared to WT or Δ*MA* individuals. On *B. oleracea*, Δ*MA* and Δ*NSP*Δ*MA* individuals grew worse compared to WT or Δ*NSP* individuals. On GSL-null *Arabidopsis*, overall growth levels among the KO lines were at a similar level.

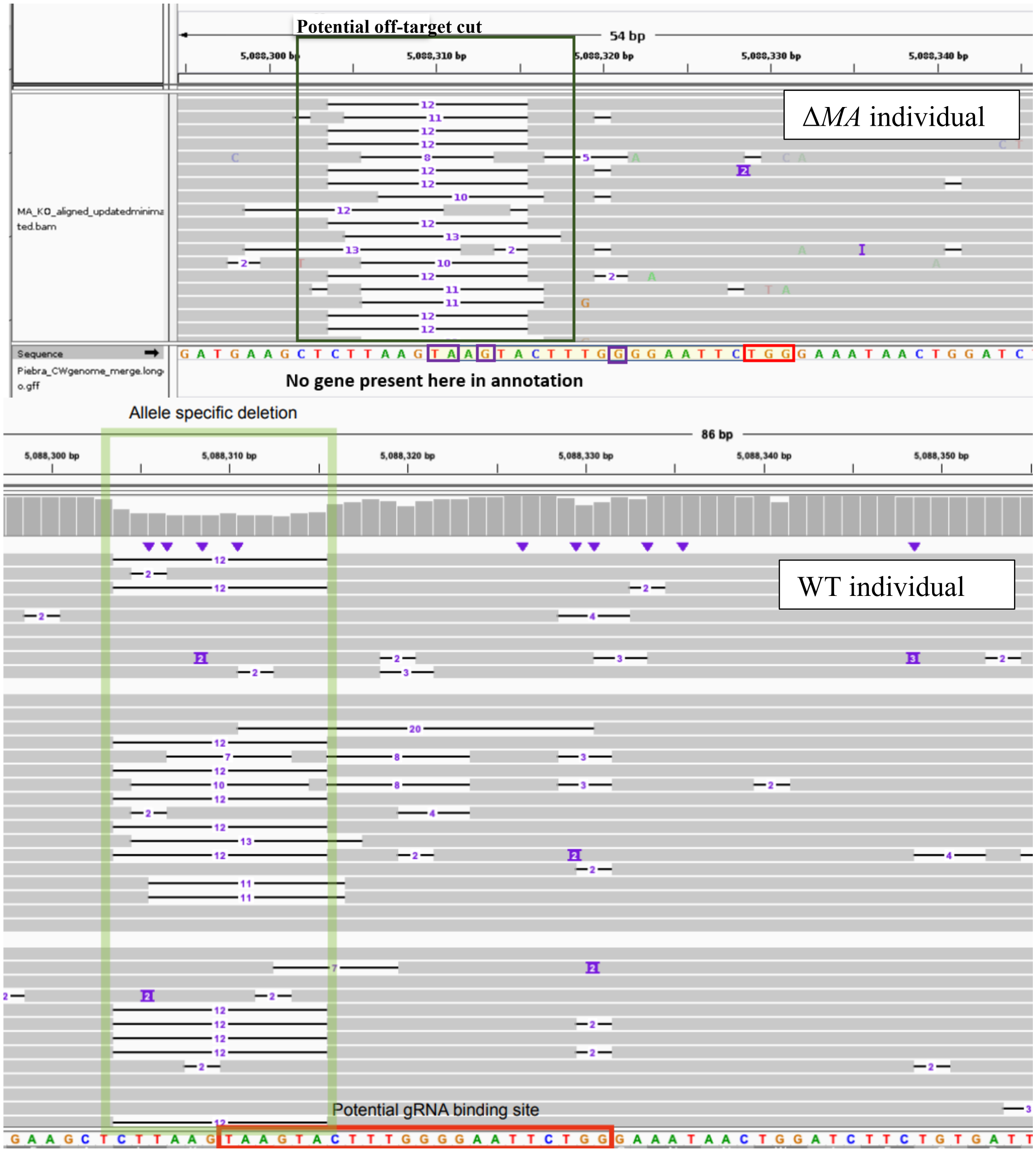

Fig. S8 (top) A potential off-target cut on contig_19 in a *ΔMA* individual, highlighted in a green box. The gRNA target site is highlighted in yellow, with mismatches identified in purple, and the PAM site in red. Note that this is on the - strand. (bottom) The same deletion (highlighted in green) can also be found in WT individuals as well, suggesting that it is NOT an off-target cut, but rather an allelic variant naturally existing within the population.

R1 (x) vs R3 (y), combined NSP fastas

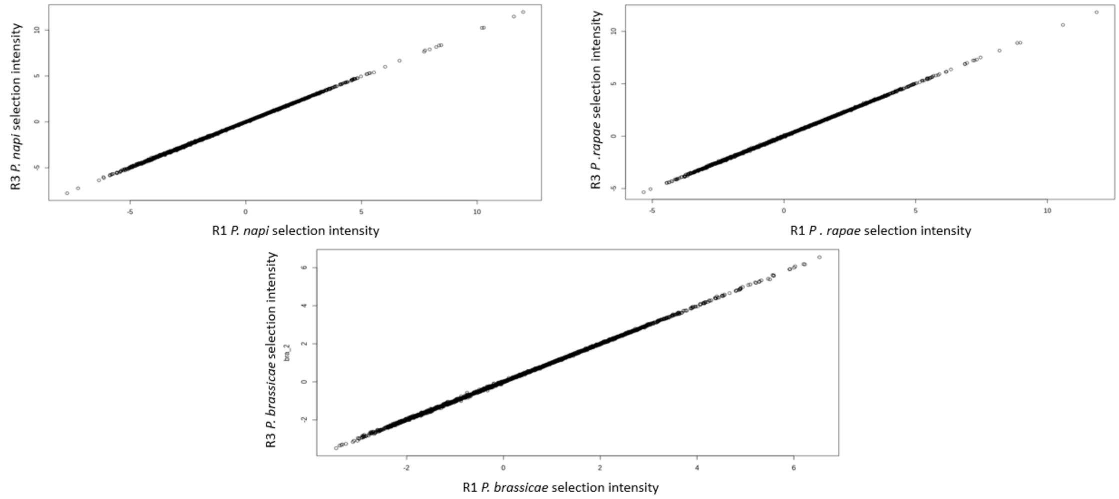

R2 (x) vs R3 (y), combined NSP fastas

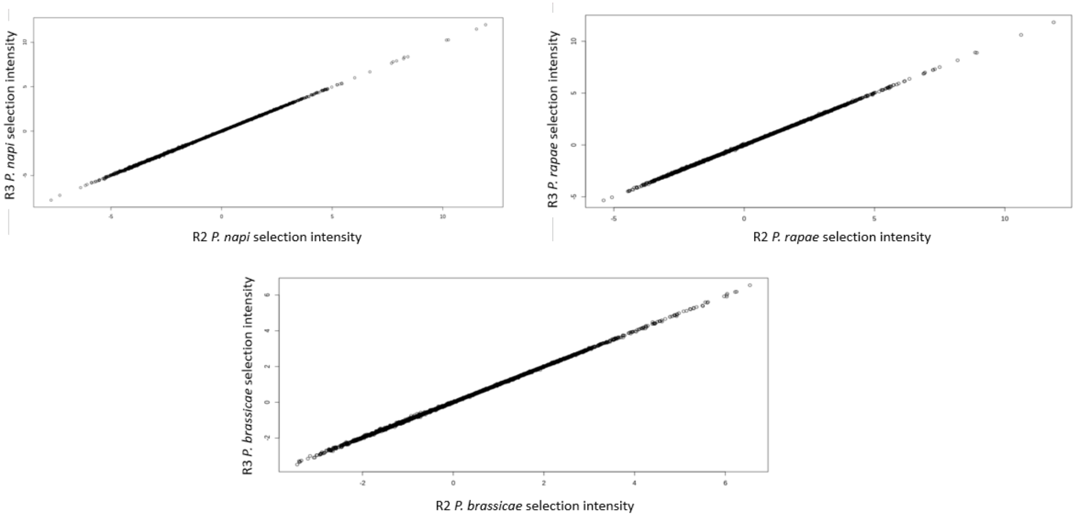

Fig. S9. Evidence for convergence of three different HDMKPRF runs with combined *NSP*-gene inputs. Here, estimated per-gene selection intensities for each run are plotted against each other, with the coordinates of a point representing the estimated intensity for a given gene in each of two runs.

Supplementary Tables

Table S1. Genome assembly stats of *Pieris brassicae*. BUSCO (v. 4.1.2) values were calculated against the lepidoptera_odb10 database (9)

	P. brassicae **genome assembly**
**Total genome size**	265,902,541
**Contigs**	87
**N50**	18,521,142
**BUSCO**	C:99.0%[S:98.8%,D:0.2%],F:0.3%,M:0.7%,n:5286
**Annotated genes**	16,334
**BUSCO on predicted genes**	C:98.0%[S:86.5%,D:11.5%],F:0.5%,M:1.5%,n:5286
**BUSCO on isoform filtered genes**	C:97.4%[S:96.7%,D:0.7%],F:0.5%,M:2.1%,n:5286

Table S2. Primers and gRNA sequences used in this study

gRNAs

NSP	GGACATAAGCAATGCCTTGG
MA	GGACTACTTTGAGGAATTCT

Primers for RT-qPCR

Name	Sequence 5'-3'	**Description**
BrNSP1_F	CGTGAGCATGCTTCCTATCA	NSP qPCR Forward
BrNSP1_R	TCCAGAGCGCCTTGTAGAGT	NSP qPCR Reverse
BrMA1_F	CAGGTTGTATCGCAAGCTGA	MA qPCR Forward
BrMA1_R	GATCGGCTAACAGGTCTTCG	MA qPCR Reverse
BrSDMA_F	GAGGGTCACGAAATCGACAT	SDMA qPCR Forward
BrSDMA_R	TTAGCTCATGGCGCTTCTTT	SDMA qPCR Reverse
BrEf1a_F	AGGAATTGCGTCGTGGTTAC	EF1a qPCR Forward
BrEf1a_R	TGGTGTGTAACCGTTGGAGA	EF1a qPCR Reverse

Primers for Genotyping

Name	Sequence 5'-3'	**Description**
NSP_start	ATGAAAGGTGTTGTAGTCTTCT TAGC	NSP CDS forward
NSP_231R	TCTTGCACTTCAGGCTCCTT	NSP exon1 reverse position 231bp
NSP_700R	CGATCGTGAGGTTGTCTAAAT ATT	NSP exon1 reverse2 position 700bp
NSP_stop	CTGGCCGTAAAGGGCA	NSP CDS reverse
Pbra_gNSP1utrF	CATCGCAACGCTATTAAATACC	NSP1 specific Forward
Pbra_gNSP1utrR	TTCCGGGCTCTTTCCAGTCT	NSP1 specific Reverse
Pbra_gNSP2utrF	GGCAAGTCATAGAAAGATCGC GA	NSP2 specific Forward
Pbra_gNSP2utrR	CAGAGATCGGTTTCCAGTCTC A	NSP2 specific Reverse

MA_start	ATGAAGACAACAATAGTGCTT CTAAG	MA CDS Forward
MA_stop	TTATTGTCCCCAGAGGGTTG	MA CDS Reverse
bra_gMA_F	AGTCTAGTATGCCTTGGGTGT G	MA RFLP Forward
MA_620R	TCTGAAGAATTCATCGTGAGC C	MA RFLP Reverse

Table S3. Genome information for *Pieris rapae* and *Pieris napi* genomes used in this study. BUSCO scores (v 4.1.2) were generated against the lepidoptera_odb10 database (10)

	***P. rapae* reference genome**	***P. napi* reference genome**
**Total genome size**	323,179,347	320,004,350
**Contigs**	2,772	49
**N50**	11,535,178	12,982,002
**BUSCO on reference genome (n = 5286)**	C:98.0%[S:92.9%,D:5.1%] F:0.9%,M:1.1%	C:99.2%[S:98.5%,D:0.7%] F:0.2%,M:0.6%
**Genome source**	Nallu et. al 2018 (30)	Darwin Tree of Life, ID: ilPieNapi4

Table S4. SNPs called by Clair3 (24) near the potential gRNA binding sites in the three KO lines generated in this study. Values in parentheses for SNP positions indicate distance from the PAM site, with negative values indicating a location upstream from the PAM site.

KO line	Contig	gRNA	Ref	Alt	gRNA mismatch	strand	PAM	position of SNP	Memo
Δ*NSP*	bctg00000001_segment0	NSP	A	ATAG	5	-	16936642	16936620 (-22)	intron of g8336
Δ*NSP*	bctg00000002_segment0	NSP	G	A	5	-	493865	493892 (27)	No gene (between g7234(448996) - g7235(518868))
Δ*NSP*	bctg00000003_segment0	NSP	G	A	5	-	20185163	20185216 (53)	No gene (between g1207 (20175288) and 1208(20198114))
Δ*NSP*	bctg00000004_segment1	NSP	CCAAGG	C	0	-	7944398	7944401 (3)	*NSP*
Δ*NSP*	bctg00000004_segment1	NSP	CCAAGG	C	0	-	7952192	7952195 (3)	*NSP*
Δ*NSP*	contig_20_segment0	NSP	T	G	5	+	1782238	1782204 (-34)	No gene (between g5523(1681904) - g5524(1894049))<br>Allelic variant (The same allele found in Δ*MA*)
Δ*NSP*	contig_20_segment0	NSP	T	C	5	+	1782238	1782205 (-33)	No gene (between g5523(1681904) - g5524(1894049))<br>Allelic variant (The same allele found in Δ*MA*)
Δ*NSP*	contig_20_segment0	NSP	A	C	5	+	1782238	1782225 (-13)	No gene (between g5523(1681904) - g5524(1894049))<br>Allelic variant (The same allele found in Δ*MA*)
Δ*NSP*	contig_20_segment0	NSP	C	T	5	+	1782238	1782232 (-6)	No gene (between g5523(1681904) - g5524(1894049))<br>Allelic variant (The same allele found in Δ*MA*)
Δ*NSP*	contig_20_segment0	NSP	C	G	5	+	1782238	1782233 (-5)	No gene (between g5523(1681904) - g5524(1894049))<br>Allelic variant (The same allele found in Δ*MA*)
Δ*NSP*	contig_20_segment0	NSP	A	G	5	+	1782238	1782238 (0)	No gene (between g5523(1681904) - g5524(1894049))<br>Allelic variant (The same allele found in Δ*MA*)
Δ*MA*	bctg00000001_segment0	MA	C	T	5	-	2367054	2367080 (26)	No BLAST hits
Δ*MA*	bctg00000004_segment1	MA	A	T	5	+	11855006	11854946 (-60)	Intron of g13918
Δ*MA*	contig_16_segment0	MA	A	AT	0	-	1438042	1438047 (5)	*MA*
Δ*MA*	contig_19_segment0	MA	TCTTAA GTAAGT A	T	4	+	5088330	5088303 (-27)	No gene present (between g9110 (5079673) and g9111/nbisL1-transcript-10317(5106320))<br>Allelic variant (The same allele found in WT)
Δ*NSP*Δ*MA*	bctg00000001_segment0	NSP	A	ATAG	5	-	16936642	16936620 (22)	g8336.t1_intron
Δ*NSP*Δ*MA*	bctg00000002_segment0	NSP	G	A	5	-	493865	493892 (27)	No Gene (between g7234(448996) - g7235(518868))

Δ*NSP*Δ*MA*	bctg00000004_segment1	NSP	CCAAGG	C	0	-	7944398	7944401 (3)	*NSP*
Δ*NSP*Δ*MA*	bctg00000004_segment1	NSP	CCAAGG	C	0	-	7952192	7952195 (3)	*NSP*
Δ*NSP*Δ*MA*	contig_122_segment0	NSP	T	A	5	-	1178419	1178376 (-43)	g8721.t1_intron
Δ*NSP*Δ*MA*	contig_20_segment0	NSP	T	G	5	+	1782238	1782204 (-34)	No Gene (between g5523(1681904) - g5524(1894049)) Allelic variant (The same allele found in Δ*MA*)
Δ*NSP*Δ*MA*	contig_20_segment0	NSP	T	C	5	+	1782238	1782205 (-33)	No Gene (between g5523(1681904) - g5524(1894049)) Allelic variant (The same allele found in Δ*MA*)
Δ*NSP*Δ*MA*	contig_20_segment0	NSP	G	C	5	+	1782238	1782225 (-13)	No Gene (between g5523(1681904) - g5524(1894049)) Allelic variant (The same allele found in Δ*MA*)
Δ*NSP*Δ*MA*	contig_20_segment0	NSP	C	T	5	+	1782238	1782232 (-6)	No Gene (between g5523(1681904) - g5524(1894049)) Allelic variant (The same allele found in Δ*MA*)
Δ*NSP*Δ*MA*	contig_20_segment0	NSP	C	G	5	+	1782238	1782233 (-5)	No Gene (between g5523(1681904) - g5524(1894049)) Allelic variant (The same allele found in Δ*MA*)
Δ*NSP*Δ*MA*	contig_20_segment0	NSP	A	G	5	+	1782238	1782238 (0)	No Gene (between g5523(1681904) - g5524(1894049)) Allelic variant (The same allele found in Δ*MA*)
Δ*NSP*Δ*MA*	contig_24_segment0	NSP	CA	C	5	-	3456941	3456925 (-16)	No Gene (between g7430(3428252) - g7431(3486693))
Δ*NSP*Δ*MA*	bctg00000001_segment0	MA	C	T	5	-	2367054	2367080 (26)	g7663.t1_intron
Δ*NSP*Δ*MA*	contig_139_segment0	MA	C	T	5	+	490158	490120 (-38)	g6546.t3_intron
Δ*NSP*Δ*MA*	contig_16_segment0	MA	A	AC	0	-	1438042	1438047 (5)	*MA*

Table S5. (a) Glucosinolate spike in feeding assay**.** Statistical values for pairwise comparisons; comparisons: the first letter of a treatment refers to the glucosinolate (B - Benzyl, I - I3M, N - NC, S - sinigrin), the second letter(s) to the mutant (M - ΔMA, N - ΔNSP, NM - ΔNSPΔMA, W - wild); Welch refers to the Welch two sample t-test, Wilcox to the Wilcoxon rank sum test with continuity correction; df - degrees of freedom; FDR - false discovery rate.

Nr	comparisons	test	t / *W*	df	FDR
1	BM-BN	Welch	-1.532	16.886	0.186
2	BM-BNM	Welch	8.344	8.523	< 0.001
3	BM-BW	Welch	-2.994	12.425	0.019
4	BN-BNM	Welch	10.687	9.611	< 0.001
5	BN-BW	Welch	-1.137	14.013	0.336
6	BNM-BW	Welch	-20.545	9.570	< 0.001
7	IM-IN	Welch	-2.173	8.984	0.089
8	IM-INM	Welch	8.790	17.562	< 0.001
9	IM-IW	*Wilcox*	*51*		0.971
10	IN-INM	Welch	4.993	8.716	0.002
11	IN-IW	*Wilcox*	*67*		0.113
12	INM-IW	*Wilcox*	*0*		< 0.001
13	NM-NN	*Wilcox*	*26*		0.320
14	NM-NNM	*Wilcox*	*32*		0.371
15	NM-NW	*Wilcox*	*16*		0.029
16	NN-NNM	*Wilcox*	*48*		0.871
17	NN-NW	*Wilcox*	*29*		0.267
18	NNM-NW	*Wilcox*	*30*		0.186
19	SM-SN	*Wilcox*	*61*		0.498
20	SM-SNM	*Wilcox*	*60*		0.001
21	SM-SW	*Wilcox*	*22*		0.056
22	SN-SNM	*Wilcox*	*60*		0.001
23	SN-SW	*Wilcox*	*8*		0.002
24	SNM-SW	*Wilcox*	*0*		0.001
25	BM-IM	Welch	1.411	9.385	0.243
26	BM-NM	*Wilcox*	*73*		0.006
27	BM-SM	*Wilcox*	*39*		0.702
28	BN-IN	Welch	0.620	15.986	0.604
29	BN-NN	*Wilcox*	*79*		0.008
30	BN-SN	*Wilcox*	*85.5*		0.015
31	BNM-INM	Welch	-11.607	11.408	< 0.001
32	BNM-NNM	Welch	-6.713	9.978	< 0.001
33	BNM-SNM	Welch	-0.225	4.164	0.868
34	BW-IW	*Wilcox*	*90*		< 0.001
35	BW-NW	*Wilcox*	*72*		0.045

Nr	comparisons	test	t / *W*	df	FDR
36	BW-SW	*Wilcox*	*21.5*		0.092
37	IM-NM	*Wilcox*	*80*		0.006
38	IM-SM	*Wilcox*	*36*		0.371
39	IN-NN	*Wilcox*	*69*		0.019
40	IN-SN	*Wilcox*	*59*		0.336
41	INM-NNM	Welch	-2.051	10.937	0.095
42	INM-SNM	Welch	13.527	10.732	< 0.001
43	IW-NW	*Wilcox*	*52*		0.935
44	IW-SW	*Wilcox*	*8*		0.002
45	NM-SM	*Wilcox*	*6*		0.002
46	NN-SN	*Wilcox*	*22*		0.095
47	NNM-SNM	Welch	6.809	9.199	< 0.001
48	NW-SW	*Wilcox*	*17*		0.020
49	BM-IN	Welch	-0.766	15.549	0.516
50	BM-INM	Welch	4.726	9.009	0.003
51	BM-IW	*Wilcox*	*58.5*		0.346
52	BM-NN	*Wilcox*	*65*		0.050
53	BM-NNM	Welch	2.569	15.367	0.035
54	BM-NW	*Wilcox*	*53*		0.604
55	BM-SN	*Wilcox*	*51*		0.702
56	BM-SNM	Welch	8.411	8.103	< 0.001
57	BM-SW	*Wilcox*	*10*		0.004
58	BN-IM	Welch	3.540	10.621	0.009
59	BN-INM	Welch	6.953	10.184	< 0.001
60	BN-IW	*Wilcox*	*87*		0.008
61	BN-NM	*Wilcox*	*84*		< 0.001
62	BN-NNM	Welch	4.337	16.981	0.001
63	BN-NW	*Wilcox*	*71*		0.164
64	BN-SM	*Wilcox*	*57*		0.682
65	BN-SNM	Welch	10.792	9.121	< 0.001
66	BN-SW	*Wilcox*	*19.5*		0.038
67	BNM-IM	Welch	-20.207	11.942	< 0.001
68	BNM-IN	Welch	-8.049	8.375	< 0.001
69	BNM-IW	*Wilcox*	*0*		0.005
70	BNM-NM	*Wilcox*	*0*		0.006
71	BNM-NN	*Wilcox*	*0*		0.006
72	BNM-NW	*Wilcox*	*0*		0.005
73	BNM-SM	*Wilcox*	*0*		0.001
74	BNM-SN	*Wilcox*	*0*		0.005
75	BNM-SW	*Wilcox*	*0*		< 0.001

Nr	comparisons	test	t / *W*	df	FDR
76	BW-IM	Welch	7.841	12.352	< 0.001
77	BW-IN	Welch	1.703	11.274	0.156
78	BW-INM	Welch	13.724	11.256	< 0.001
79	BW-NM	*Wilcox*	*79*		0.001
80	BW-NN	*Wilcox*	*78*		0.001
81	BW-NNM	Welch	7.081	15.951	< 0.001
82	BW-SM	*Wilcox*	*65*		0.154
83	BW-SN	*Wilcox*	*90*		< 0.001
84	BW-SNM	Welch	21.354	8.339	< 0.001
85	IM-NN	*Wilcox*	*66*		0.131
86	IM-NNM	Welch	2.155	11.637	0.082
87	IM-NW	*Wilcox*	*56*		0.720
88	IM-SN	*Wilcox*	*42*		0.631
89	IM-SNM	Welch	22.829	10.286	< 0.001
90	IM-SW	*Wilcox*	*6*		0.001
91	IN-NM	*Wilcox*	*76*		0.002
92	IN-NNM	Welch	3.132	13.843	0.014
93	IN-NW	*Wilcox*	*57*		0.411
94	IN-SM	*Wilcox*	*46*		0.971
95	IN-SNM	Welch	8.088	8.073	< 0.001
96	IN-SW	*Wilcox*	*15*		0.023
97	INM-NM	*Wilcox*	*44*		0.971
98	INM-NN	*Wilcox*	*24*		0.131
99	INM-NW	*Wilcox*	*10*		0.004
100	INM-SM	*Wilcox*	*0*		< 0.001
101	INM-SN	*Wilcox*	*0*		< 0.001
102	INM-SW	*Wilcox*	*0*		< 0.001
103	IW-NM	*Wilcox*	*80*		0.006
104	IW-NN	*Wilcox*	*68*		0.095
105	IW-NNM	*Wilcox*	*73.5*		0.116
106	IW-SM	*Wilcox*	*34*		0.309
107	IW-SN	*Wilcox*	*30*		0.186
108	IW-SNM	*Wilcox*	*60*		0.001
109	NM-SN	*Wilcox*	*10*		0.006
110	NM-SNM	*Wilcox*	*54*		0.001
111	NM-SW	*Wilcox*	*2*		< 0.001
112	NN-SM	*Wilcox*	*15*		0.023
113	NN-SNM	*Wilcox*	*54*		0.001
114	NN-SW	*Wilcox*	*4.5*		0.003
115	NNM-SM	*Wilcox*	*14*		0.010

Nr	comparisons	test	t / *W*	df	FDR
116	NNM-SN	*Wilcox*	*26*		0.109
117	NNM-SW	*Wilcox*	*3*		< 0.001
118	NW-SM	*Wilcox*	*36.5*		0.379
119	NW-SN	*Wilcox*	*40.5*		0.556
120	NW-SNM	*Wilcox*	*60*		0.001

(b) Wild Brassicaceae feeding assay. Statistical values for pairwise comparisons; comparisons: the first letter of a treatment refers to the glucosinolate (Bj - B. juncea, Bo - B. oleracea, N - NC (Arabidopsis null-GSL mutant : mybcyp), Tj - T. majus), the second letter(s) to the mutant (M - ΔMA, N - ΔNSP, NM - ΔNSPΔMA, W - wild); Welch refers to the Welch two sample t-test, Wilcox to the Wilcoxon rank sum test with continuity correction; df - degrees of freedom; FDR - false discovery rate.

Nr	comparisons	test	t / *W*	df	FDR
1	BjM-BjN	Welch	-16.0	12.697	< 0.001
2	BjM-BjNM	Welch	59.8	11.462	< 0.001
3	BjM-BjW	*Wilcox*	12.0		0.001
4	BjN-BjNM	Welch	5.3	10.057	< 0.001
5	BjN-BjW	*Wilcox*	131.0		< 0.001
6	BjNM-BjW	*Wilcox*	144.0		< 0.001
7	BoM-BoN	Welch	9.0	24.463	< 0.001
8	BoM-BoNM	Welch	0.7	33.722	0.510
9	BoM-BoW	*Wilcox*	400.0		< 0.001
10	BoN-BoNM	Welch	8.8	29.729	< 0.001
11	BoN-BoW	*Wilcox*	201.0		0.989
12	BoNM-BoW	*Wilcox*	400.0		< 0.001
13	NM-NN	*Wilcox*	114.0		0.061
14	NM-NNM	Welch	1.9	36.917	0.072
15	NM-NW	Welch	0.4	36.579	0.748
16	NN-NNM	*Wilcox*	176.5		0.946
17	NN-NW	*Wilcox*	246.5		0.060
18	NNM-NW	Welch	2.5	37.973	0.019
19	TjM-TjN	Welch	5.1	28.949	< 0.001
20	TjM-TjNM	Welch	4.5	16.375	< 0.001
21	TjM-TjW	Welch	11.5	22.843	< 0.001
22	TjN-TjNM	Welch	15.1	17.815	< 0.001
23	TjN-TjW	Welch	7.8	30.210	< 0.001
24	TjNM-TjW	Welch	35.9	21.025	< 0.001
25	BjM-BoM	Welch	12.2	29.676	< 0.001
26	BjM-NM	Welch	19.4	27.409	< 0.001
27	BjM-TjM	Welch	20.0	21.877	< 0.001

28	BjN-BoN	Welch	-13.8	27.986	< 0.001
29	BjN-NN	*Wilcox*	23.0		< 0.001
30	BjN-TjN	Welch	-3.6	16.547	0.003
31	BjNM-BoNM	Welch	-23.3	19.271	< 0.001
32	BjNM-NNM	Welch	-37.3	20.182	< 0.001
33	BjNM-TjNM	Welch	-6.7	27.561	< 0.001
34	BjW-BoW	*Wilcox*	20.0		< 0.001
35	BjW-NW	*Wilcox*	215.0		< 0.001
36	BjW-TjW	*Wilcox*	131.0		0.712
37	BoM-NM	Welch	5.4	36.612	< 0.001
38	BoM-TjM	Welch	11.8	24.745	< 0.001
39	BoN-NN	*Wilcox*	360.0		< 0.001
40	BoN-TjN	Welch	13.2	27.786	< 0.001
41	BoNM-NNM	Welch	4.8	27.184	< 0.001
42	BoNM-TjNM	Welch	22.0	19.739	< 0.001
43	BoW-NW	*Wilcox*	400.0		< 0.001
44	BoW-TjW	*Wilcox*	400.0		< 0.001
45	NM-TjM	Welch	8.9	22.064	< 0.001
46	NN-TjN	*Wilcox*	227.0		0.046
47	NNM-TjNM	Welch	34.2	22.232	< 0.001
48	NW-TjW	Welch	-4.8	35.637	< 0.001
49	BjM-BoN	Welch	-3.3	22.767	0.003
50	BjM-BoNM	Welch	10.3	28.432	< 0.001
51	BjM-BoW	*Wilcox*	19.0		< 0.001
52	BjM-NN	*Wilcox*	216.0		< 0.001
53	BjM-NNM	Welch	22.8	24.304	< 0.001
54	BjM-NW	Welch	20.8	23.796	< 0.001
55	BjM-TjN	Welch	19.0	26.916	< 0.001
56	BjM-TjNM	Welch	57.0	12.272	< 0.001
57	BjM-TjW	Welch	14.2	28.348	< 0.001
58	BjN-BoM	Welch	-9.2	13.908	< 0.001
59	BjN-BoNM	Welch	-8.0	18.103	< 0.001
60	BjN-BoW	*Wilcox*	0.0		< 0.001
61	BjN-NM	Welch	-6.6	12.624	< 0.001
62	BjN-NNM	Welch	-5.9	11.849	< 0.001
63	BjN-NW	Welch	-6.9	11.751	< 0.001
64	BjN-TjM	Welch	0.7	21.745	0.510
65	BjN-TjNM	Welch	4.6	10.156	0.001
66	BjN-TjW	Welch	-8.8	12.985	< 0.001

67	BjNM-BoM	Welch	-34.9	19.568	< 0.001
68	BjNM-BoN	Welch	-23.1	19.084	< 0.001
69	BjNM-BoW	*Wilcox*	0.0		< 0.001
70	BjNM-NM	Welch	-34.0	19.840	< 0.001
71	BjNM-NN	*Wilcox*	0.0		< 0.001
72	BjNM-NW	Welch	-41.8	20.246	< 0.001
73	BjNM-TjM	Welch	-5.3	16.137	< 0.001
74	BjNM-TjN	Welch	-16.4	17.298	< 0.001
75	BjNM-TjW	Welch	-38.3	19.741	< 0.001
76	BjW-BoM	*Wilcox*	106.0		0.631
77	BjW-BoN	*Wilcox*	22.0		< 0.001
78	BjW-BoNM	*Wilcox*	129.0		0.760
79	BjW-NM	*Wilcox*	214.0		< 0.001
80	BjW-NN	*Wilcox*	211.0		< 0.001
81	BjW-NNM	*Wilcox*	234.5		< 0.001
82	BjW-TjM	*Wilcox*	204.0		< 0.001
83	BjW-TjN	*Wilcox*	215.0		< 0.001
84	BjW-TjNM	*Wilcox*	228.0		< 0.001
85	BoM-NN	*Wilcox*	344.0		< 0.001
86	BoM-NNM	Welch	7.4	33.765	< 0.001
87	BoM-NW	Welch	5.4	33.264	< 0.001
88	BoM-TjN	Welch	8.2	32.690	< 0.001
89	BoM-TjNM	Welch	32.9	20.553	< 0.001
90	BoM-TjW	Welch	1.0	37.342	0.361
91	BoN-NM	Welch	11.7	22.725	< 0.001
92	BoN-NNM	Welch	12.6	21.649	< 0.001
93	BoN-NW	Welch	11.7	21.512	< 0.001
94	BoN-TjM	Welch	15.6	33.154	< 0.001
95	BoN-TjNM	Welch	22.4	19.229	< 0.001
96	BoN-TjW	Welch	9.6	23.220	< 0.001
97	BoNM-NM	Welch	3.4	30.030	0.002
98	BoNM-NN	*Wilcox*	327.0		< 0.001
99	BoNM-NW	Welch	3.3	26.796	0.003
100	BoNM-TjM	Welch	10.1	31.455	< 0.001
101	BoNM-TjN	Welch	6.3	35.916	< 0.001
102	BoNM-TjW	Welch	0.0	31.200	0.988
103	BoW-NM	*Wilcox*	400.0		< 0.001
104	BoW-NN	*Wilcox*	360.0		< 0.001
105	BoW-NNM	*Wilcox*	400.0		< 0.001
106	BoW-TjM	*Wilcox*	340.0		< 0.001

107	BoW-TjN	*Wilcox*	360.0		< 0.001
108	BoW-TjNM	*Wilcox*	380.0		< 0.001
109	NM-TjN	Welch	4.3	29.007	< 0.001
110	NM-TjNM	Welch	31.5	21.297	< 0.001
111	NM-TjW	Welch	-4.7	37.847	< 0.001
112	NN-TjM	*Wilcox*	283.0		< 0.001
113	NN-TjNM	*Wilcox*	342.0		< 0.001
114	NN-TjW	*Wilcox*	20.5		< 0.001
115	NNM-TjM	Welch	8.1	20.338	< 0.001
116	NNM-TjN	Welch	3.1	25.993	0.005
117	NNM-TjW	Welch	-6.9	36.053	< 0.001
118	NW-TjM	Welch	9.3	20.116	< 0.001
119	NW-TjN	Welch	4.8	25.575	< 0.001
120	NW-TjNM	Welch	38.5	22.406	< 0.001

(c) Wild Brassicaceae feeding assay (replicate). Statistical values for pairwise comparisons; comparisons: the first letter of a treatment refers to the glucosinolate (Bj - B. juncea, Bo - B. oleracea, N - NC (Arabidopsis null-GSL mutant : mybcyp)), the second letter(s) to the mutant (M - ΔMA, N - ΔNSP, NM - ΔNSPΔMA, W - wild); Welch refers to the Welch two sample t-test, Wilcox to the Wilcoxon rank sum test with continuity correction; df - degrees of freedom; FDR - false discovery rate.

**Nr**	**comparisons**	**test**	**t / *W***	**df**	**FDR**
1	BjM-BjN	*Wilcox*	163.00		**0.005**
2	BjM-BjNM	Welch	34.28	26.98	**< 0.001**
3	BjM-BjW	Welch	-10.83	34.90	**< 0.001**
4	BjN-BjNM	*Wilcox*	625.00		**< 0.001**
5	BjN-BjW	*Wilcox*	7.00		**< 0.001**
6	BjNM-BjW	Welch	29.16	24.72	**< 0.001**
7	BoM-BoN	Welch	9.70	26.51	**< 0.001**
8	BoM-BoNM	*Wilcox*	174.50		0.253
9	BoM-BoW	*Wilcox*	0.00		**< 0.001**
10	BoN-BoNM	*Wilcox*	390.50		**< 0.001**
11	BoN-BoW	*Wilcox*	74.50		**0.001**
12	BoNM-BoW	*Wilcox*	420.00		**< 0.001**
13	NM-NN	Welch	1.13	29.62	0.279
14	NM-NNM	Welch	5.19	29.99	**< 0.001**
15	NM-NW	Welch	2.52	28.18	**0.021**
16	NN-NNM	Welch	6.02	29.75	**< 0.001**
17	NN-NW	Welch	3.41	29.38	**0.002**
18	NNM-NW	Welch	1.99	28.44	0.063
19	BjM-BoM	Welch	17.21	33.53	**< 0.001**

20	BjM-NM	Welch	10.75	34.28	**< 0.001**
21	BjN-BoN	*Wilcox*	208.50		0.354
22	BjN-NN	*Wilcox*	273.50		0.058
23	BjNM-BoNM	*Wilcox*	19.50		**< 0.001**
24	BjNM-NNM	Welch	-8.89	16.39	**< 0.001**
25	BjW-BoW	*Wilcox*	423.50		**0.001**
26	BjW-NW	Welch	18.11	38.73	**< 0.001**
27	BoM-NM	Welch	-2.35	20.39	**0.034**
28	BoN-NN	Welch	3.88	30.98	**0.001**
29	BoNM-NNM	*Wilcox*	215.00		0.091
30	BoW-NW	*Wilcox*	335.00		**< 0.001**
31	BjM-BoN	Welch	-5.47	41.84	**< 0.001**
32	BjM-BoNM	*Wilcox*	500.00		**< 0.001**
33	BjM-BoW	*Wilcox*	214.00		0.299
34	BjM-NN	Welch	-8.86	31.62	**< 0.001**
35	BjM-NNM	Welch	16.13	33.81	**< 0.001**
36	BjM-NW	Welch	11.77	28.19	**< 0.001**
37	BjN-BoM	*Wilcox*	321.00		**< 0.001**
38	BjN-BoNM	*Wilcox*	458.00		**< 0.001**
39	BjN-BoW	*Wilcox*	94.00		**< 0.001**
40	BjN-NM	*Wilcox*	309.50		**0.004**
41	BjN-NNM	*Wilcox*	374.00		**< 0.001**
42	BjN-NW	*Wilcox*	332.00		**< 0.001**
43	BjNM-BoM	Welch	28.99	19.72	**< 0.001**
44	BjNM-BoN	Welch	25.36	21.15	**< 0.001**
45	BjNM-BoW	*Wilcox*	525.00		**< 0.001**
46	BjNM-NM	Welch	16.31	16.45	**< 0.001**
47	BjNM-NN	Welch	16.12	16.15	**< 0.001**
48	BjNM-NW	Welch	9.55	15.86	**< 0.001**
49	BjW-BoM	Welch	-20.87	26.75	**< 0.001**
50	BjW-BoN	Welch	-14.18	35.28	**< 0.001**
51	BjW-BoNM	*Wilcox*	500.00		**< 0.001**
52	BjW-NM	Welch	-17.50	35.75	**< 0.001**
53	BjW-NN	Welch	-16.19	37.32	**< 0.001**
54	BjW-NNM	Welch	21.01	36.05	**< 0.001**
55	BoM-NN	Welch	3.55	19.37	**0.003**
56	BoM-NNM	Welch	4.38	20.19	**< 0.001**
57	BoM-NW	Welch	1.15	18.31	0.279
58	BoN-NM	Welch	5.39	32.69	**< 0.001**

59	BoN-NNM	Welch	10.72	32.42	**< 0.001**
60	BoN-NW	Welch	7.21	28.29	**< 0.001**
61	BoNM-NM	*Wilcox*	258.00		**0.002**
62	BoNM-NN	*Wilcox*	269.00		**0.001**
63	BoNM-NW	*Wilcox*	161.00		0.987
64	BoW-NM	*Wilcox*	5.00		**< 0.001**
65	BoW-NN	*Wilcox*	13.50		**< 0.001**
66	BoW-NNM	*Wilcox*	336.00		**< 0.001**

Table S6. Selection intensities for *NSP, MA,* and *SDMA* in three *Pieris* species. Selection coefficients and p-values for *MA* and *NSP* were calculated against a set of 4790 single-copy orthologs with the HDMKPRF test (33). For all *MA* and *NSP* genes (except MA in *P. brassicae*), significant positive selection was detected. No positive selection was detected for *SDMA*. Selection intensities reported below are the median values from three HDMKPRF runs; for a full set of results, see *SI Data*, File 1.

**Species**	**Gene**	**Selection intensity**	**p-value (coefficient > 0)**	**Selection detected?**
P. brassicae	*NSP*	1.675	0.0011	Yes, positive
P. brassicae	*MA*	0.598	0.1283	No
P. brassicae	*SDMA*	-0.301	0.3639	No
P. napi	*NSP*	2.029	0.0004	Yes, positive
P. napi	*MA*	1.176	0.0144	Yes, positive
P. napi	*SDMA*	0.232	0.5603	No
P. rapae	*NSP*	2.665	0	Yes, positive
P. rapae	*MA*	1.641	0.0010	Yes, positive
P. rapae	*SDMA*	-0.531	0.2852	No

Table S7. Full HDMKPRF test results for *NSP*, *MA,* and *SDMA* in three species of *Pieris*. A value of “1a” in the NSP column indicates that only the first copy of *NSP* present in each species was assessed in the analyses. Conversely, a value of “Combo” indicates that *NSP* sequences from all gene copies were assessed, provided multiple copies were present in the species of interest.

Species	Gene	Run	NSP	Selection intensity	p-value	Selection detected?
P. brassicae	NSP	1	1a	1.965	0.0004	Yes, positive
P. brassicae	NSP	2	1a	1.936	0.0002	Yes, positive
P. brassicae	NSP	3	1a	1.958	0.0001	Yes, positive
P. brassicae	NSP	1	Combo	1.668	0.0013	Yes, positive
P. brassicae	NSP	2	Combo	1.683	0.0012	Yes, positive
P. brassicae	NSP	3	Combo	1.675	0.0011	Yes, positive
P. brassicae	MA	1	1a	0.614	0.1155	No
P. brassicae	MA	2	1a	0.598	0.13	No
P. brassicae	MA	3	1a	0.601	0.1215	No
P. brassicae	MA	1	Combo	0.598	0.1283	No
P. brassicae	MA	2	Combo	0.597	0.1279	No
P. brassicae	MA	3	Combo	0.616	0.1158	No
P. brassicae	SDMA	1	1a	-0.310906	0.3632	No
P. brassicae	SDMA	2	1a	-0.290038	0.3718	No
P. brassicae	SDMA	3	1a	-0.30867	0.3608	No
P. brassicae	SDMA	1	Combo	-0.300569	0.3639	No
P. brassicae	SDMA	2	Combo	-0.291558	0.368	No
P. brassicae	SDMA	3	Combo	-0.312892	0.3586	No
P. napi	NSP	1	1a	2.495	0	Yes, positive
P. napi	NSP	2	1a	2.475	0	Yes, positive
P. napi	NSP	3	1a	2.474	0	Yes, positive
P. napi	NSP	1	Combo	2.028	0	Yes, positive
P. napi	NSP	2	Combo	2.029	0.0004	Yes, positive
P. napi	NSP	3	Combo	2.031	0.0002	Yes, positive
P. napi	MA	1	1a	1.178	0.0131	Yes, positive

P. napi	MA	2	1a	1.162	0.0129	Yes, positive
P. napi	MA	3	1a	1.178	0.0123	Yes, positive
P. napi	MA	1	Combo	1.176	0.0144	Yes, positive
P. napi	MA	2	Combo	1.167	0.0137	Yes, positive
P. napi	MA	3	Combo	1.181	0.0103	Yes, positive
P. napi	SDMA	1	1a	0.238692	0.5631	No
P. napi	SDMA	2	1a	0.249052	0.5696	No
P. napi	SDMA	3	1a	0.226153	0.5592	No
P. napi	SDMA	1	Combo	0.231775	0.5603	No
P. napi	SDMA	2	Combo	0.236167	0.565	No
P. napi	SDMA	3	Combo	0.228853	0.5636	No
P. rapae	NSP	1	1a	3.133	0	Yes, positive
P. rapae	NSP	2	1a	3.111	0	Yes, positive
P. rapae	NSP	3	1a	3.112	0	Yes, positive
P. rapae	NSP	1	Combo	2.655	0	Yes, positive
P. rapae	NSP	2	Combo	2.665	0	Yes, positive
P. rapae	NSP	3	Combo	2.658	0.0001	Yes, positive
P. rapae	MA	1	1a	1.644	0.0009	Yes, positive
P. rapae	MA	2	1a	1.634	0.001	Yes, positive
P. rapae	MA	3	1a	1.638	0.0007	Yes, positive
P. rapae	MA	1	Combo	1.648	0.0011	Yes, positive
P. rapae	MA	2	Combo	1.639	0.0016	Yes, positive
P. rapae	MA	3	Combo	1.641	0.001	Yes, positive
P. rapae	SDMA	1	1a	-0.543715	0.2802	No
P. rapae	SDMA	2	1a	-0.503734	0.2948	No
P. rapae	SDMA	3	1a	-0.554341	0.2795	No
P. rapae	SDMA	1	Combo	-0.516834	0.292	No
P. rapae	SDMA	2	Combo	-0.533243	0.2855	No
P. rapae	SDMA	3	Combo	-0.531364	0.2852	No

Table S8. Counts and percentages of single-copy ortholog genes identified as under significant selection by HDMKPRF by species.

Species	# SCO genes under significant selection	% of total (n=4789) SCO genes under significant selection
P. napi	**1365**	**28.50**
P. rapae	**1516**	**31.66**
P. brassicae	**1355**	**28.29**

SI References

1. R. R. Wick, L. M. Judd, K. E. Holt, Performance of neural network basecalling tools for Oxford Nanopore sequencing. *Genome Biol.* **20**, 129 (2019).

2. M. Kolmogorov, J. Yuan, Y. Lin, P. Pevzner, Assembly of Long Error-Prone Reads Using Repeat Graphs. *Nat. Biotechnol.* **37**, 540–546 (2019).

3. Y. Chen, *et al.*, Efficient assembly of nanopore reads via highly accurate and intact error correction. *Nat. Commun.* **12**, 60 (2021).

4. R. Vaser, I. Sović, N. Nagarajan, M. Šikić, Fast and accurate de novo genome assembly from long uncorrected reads. *Genome Res.* **27**, 737–746 (2017).

5. M. J. Roach, S. A. Schmidt, A. R. Borneman, Purge Haplotigs: allelic contig reassignment for third-gen diploid genome assemblies. *BMC Bioinformatics* **19**, 460 (2018).

6. M. Chakraborty, J. G. Baldwin-Brown, A. D. Long, J. J. Emerson, Contiguous and accurate de novo assembly of metazoan genomes with modest long read coverage. *Nucleic Acids Res.* **44**, e147 (2016).

7. R. L. Warren, *et al.*, ntEdit: scalable genome sequence polishing. *Bioinformatics* **35**, 4430–4432 (2019).

8. F. A. Simão, R. M. Waterhouse, P. Ioannidis, E. V. Kriventseva, E. M. Zdobnov, BUSCO: assessing genome assembly and annotation completeness with single-copy orthologs. *Bioinformatics* **31**, 3210–3212 (2015).

9. M. Manni, M. R. Berkeley, M. Seppey, F. A. Simão, E. M. Zdobnov, BUSCO Update: Novel and Streamlined Workflows along with Broader and Deeper Phylogenetic Coverage for Scoring of Eukaryotic, Prokaryotic, and Viral Genomes. *Mol. Biol. Evol.* **38**, 4647–4654 (2021).

10. E. V. Kriventseva, *et al.*, OrthoDB v10: sampling the diversity of animal, plant, fungal, protist, bacterial and viral genomes for evolutionary and functional annotations of orthologs. *Nucleic Acids Res.* **47**, D807–D811 (2019).

11. J. M. Flynn, *et al.*, RepeatModeler2 for automated genomic discovery of transposable element families. *Proc. Natl. Acad. Sci.* **117**, 9451–9457 (2020).

12. Z. Bao, S. R. Eddy, Automated De Novo Identification of Repeat Sequence Families in Sequenced Genomes. *Genome Res.* **12**, 1269–1276 (2002).

13. A. L. Price, N. C. Jones, P. A. Pevzner, De novo identification of repeat families in large genomes. *Bioinformatics* **21**, i351–i358 (2005).

14. G. Benson, Tandem repeats finder: a program to analyze DNA sequences. *Nucleic Acids Res.* **27**, 573–580 (1999).

15. A. Dobin, *et al.*, STAR: ultrafast universal RNA-seq aligner. *Bioinformatics* **29**, 15–21 (2013).

16. A. R. Quinlan, I. M. Hall, BEDTools: a flexible suite of utilities for comparing genomic features. *Bioinforma. Oxf. Engl.* **26**, 841–842 (2010).

17. J. Dainat, AGAT: Another Gff Analysis Toolkit to handle annotations in any GTF/GFF format. (2021).

18. J. D. Sander, P. Zaback, J. K. Joung, D. F. Voytas, D. Dobbs, Zinc Finger Targeter (ZiFiT): an engineered zinc finger/target site design tool. *Nucleic Acids Res.* **35**, W599-605 (2007).

19. J. D. Sander, *et al.*, ZiFiT (Zinc Finger Targeter): an updated zinc finger engineering tool. *Nucleic Acids Res.* **38**, W462-468 (2010).

20. H. Jiang, W. H. Wong, SeqMap: mapping massive amount of oligonucleotides to the genome. *Bioinformatics* **24**, 2395–2396 (2008).

21. X. Guo, *et al.*, Efficient RNA/Cas9-mediated genome editing in Xenopus tropicalis. *Development* **141**, 707–714 (2014).

22. H. Li, Minimap2: pairwise alignment for nucleotide sequences. *Bioinformatics* **34**, 3094–3100 (2018).

23. H. Li, New strategies to improve minimap2 alignment accuracy. *Bioinformatics* **37**, 4572–4574 (2021).

24. Z. Zheng, *et al.*, Symphonizing pileup and full-alignment for deep learning-based long-read variant calling. 2021.12.29.474431 (2021).

25. J. C. Pinheiro, D. M. Bates, *Mixed-Effects Models in S and S-PLUS*, 1st Ed. (Springer New York, 2000) (September 12, 2022).

26. J. Pinheiro, D. Bates, R Core Team, nlme: Linear and Nonlinear Mixed Effects Models (2022) (September 12, 2022).

27. A. F. Zuur, E. N. Ieno, N. Walker, A. A. Saveliev, G. M. Smith, *Mixed effects models and extensions in ecology with R* (Springer, 2009) https:/doi.org/10.1007/978-0-387-87458-6 (September 12, 2022).

28. M. J. Crawley, *The R book*, Second edition (Wiley, 2013).

29. R Core Team, R: A language and environment for statistical computing. (2020).

30. S. Nallu, *et al.*, The molecular genetic basis of herbivory between butterflies and their host plants. *Nat. Ecol. Evol.* **2**, 1418–1427 (2018).

31. T. Brůna, K. J. Hoff, A. Lomsadze, M. Stanke, M. Borodovsky, BRAKER2: automatic eukaryotic genome annotation with GeneMark-EP+ and AUGUSTUS supported by a protein database. *NAR Genomics Bioinforma.* **3** (2021).

32. H. Z. Girgis, Red: an intelligent, rapid, accurate tool for detecting repeats de-novo on the genomic scale. *BMC Bioinformatics* **16**, 227 (2015).

33. S. Zhao, *et al.*, Identifying Lineage-Specific Targets of Natural Selection by a Bayesian Analysis of Genomic Polymorphisms and Divergence from Multiple Species. *Mol. Biol. Evol.* **36**, 1302–1315 (2019).

34. J. H. McDonald, M. Kreitman, Adaptive protein evolution at the Adh locus in Drosophila. *Nature* **351**, 652–654 (1991).

35. C. D. Bustamante, *et al.*, The cost of inbreeding in Arabidopsis. *Nature* **416**, 531–534 (2002).

36. C. D. Bustamante, *et al.*, Natural selection on protein-coding genes in the human genome. *Nature* **437**, 1153–1157 (2005).

37. Y. Okamura, *et al.*, Molecular signatures of selection associated with host plant differences in Pieris butterflies. *Mol. Ecol.* **28**, 4958–4970 (2019).

38. P. P. Edger, *et al.*, The butterfly plant arms-race escalated by gene and genome duplications. *Proc. Natl. Acad. Sci.* **112**, 8362–8366 (2015).

39. Z. Yang, PAML 4: phylogenetic analysis by maximum likelihood. *Mol. Biol. Evol.* **24**, 1586–1591 (2007).

40. S. Kryazhimskiy, J. B. Plotkin, The Population Genetics of dN/dS. *PLOS Genet.* **4**, e1000304 (2008).

41. D. C. Jeffares, B. Tomiczek, V. Sojo, M. dos Reis, "A Beginners Guide to Estimating the Non-synonymous to Synonymous Rate Ratio of all Protein-Coding Genes in a Genome" in *Parasite Genomics Protocols*, Methods in Molecular Biology., C. Peacock, Ed. (Springer, 2015), pp. 65–90.

42. M. W. Hahn, *Molecular Population Genetics*, 1st Ed. (Oxford University Press, 2019).

43. D. J. Begun, *et al.*, Population Genomics: Whole-Genome Analysis of Polymorphism and Divergence in Drosophila simulans. *PLOS Biol.* **5**, e310 (2007).

44. Y. F. Li, J. C. Costello, A. K. Holloway, M. W. Hahn, "Reverse Ecology" and the Power of Population Genomics. *Evolution* **62**, 2984–2994 (2008).

45. R. Kofler, *et al.*, PoPoolation: A Toolbox for Population Genetic Analysis of Next Generation Sequencing Data from Pooled Individuals. *PLOS ONE* **6**, e15925 (2011).

46. N. L. P. Keehnen, J. Hill, S. Nylin, C. W. Wheat, Microevolutionary selection dynamics acting on immune genes of the green-veined white butterfly, Pieris napi. *Mol. Ecol.* **27**, 2807–2822 (2018).

47. V. Nolte, R. V. Pandey, R. Kofler, C. Schlötterer, Genome-wide patterns of natural variation reveal strong selective sweeps and ongoing genomic conflict in Drosophila mauritiana. *Genome Res.* **23**, 99–110 (2013).

48. M. W. A. Pfaffl, new mathematical model for relative quantification in real-time RT-PCR. *Nucleic Acids Res.* **29,** e45 (2001).
